# Circulating CD8 T cells from patients with mild-to-moderate psoriasis are functionally impaired

**DOI:** 10.3389/fimmu.2025.1585378

**Published:** 2025-05-05

**Authors:** Yiqiao Chen, Chiara Tontini, Isabella Tosi, Jonathan N. Barker, Paola Di Meglio, Christopher E. M. Griffiths, Rajia Bahri, Silvia Bulfone-Paus

**Affiliations:** ^1^ Lydia Becker Institute of Immunology and Inflammation, School of Biological Sciences, Faculty of Biology, Medicine and Health, University of Manchester, Manchester, United Kingdom; ^2^ St. John’s Institute of Dermatology, School of Basic and Medical Biosciences, King’s College London, London, United Kingdom; ^3^ Dermatology Centre, Manchester Academic Health Science Centre, Northern Care Alliance NHS Foundation Trust, Manchester, United Kingdom; ^4^ Department of Dermatology, King’s College Hospital, King’s College London, London, United Kingdom

**Keywords:** flow cytometry, CD8 T cells, multiparametric analysis, unsupervised clustering, immunophenotyping, peripheral blood mononuclear cells, psoriasis, PASI

## Abstract

**Background:**

Psoriasis is a systemic, immune-mediated, inflammatory skin disease in which T cells have been found to play a significant role. The phenotypic and functional properties of circulating CD8 T cells in the pathogenesis of the disease are still ill-defined.

**Objective:**

This study aimed to assess changes in the phenotype, activation status and mediator release of CD8 T cells in the peripheral blood of patients with mild-to-moderate psoriasis.

**Methods:**

Peripheral blood mononuclear cells from patients with mild-to-moderate psoriasis and healthy individuals were used to investigate the CD8 T cell immune phenotype and mediator release upon *in vitro* TCR-independent (phorbol 12-myristate 13-acetate (PMA) plus ionomycin (ION)) or TCR-dependent (anti-CD3/CD28) activation by flow cytometry.

**Results:**

Patients with psoriasis exhibited reduced circulating CD8 memory T cell frequency compared to healthy controls. Additionally, although CD8 T cell subsets showed similar levels of the skin homing marker CCR4, they demonstrated a significant upregulation of B- and T-lymphocyte attenuator (BTLA) expression compared to healthy individuals. Upon CD8 T cell activation, IL-17A and IL-17F were expressed at low and comparable levels in psoriasis patients and healthy controls. In contrast, CD69, IFNγ, and Granzyme B were significantly decreased in anti-CD3/CD28-activated CD8 T cell subsets. PASI scores positively correlated with IFNγ-producing CD8 T memory cells and negatively with TNF-producing CD8- T cell subsets.

**Conclusion:**

Patients with mild-to-moderate psoriasis showed a significant decrease in CD8 T memory cells and reduced release of cytotoxic mediators by CD8 T cells. Thus, this indicates that psoriasis impacts the functionality of circulating CD8 T cells.

## 1 Introduction

Psoriasis is a common, systemic and chronic immune-modulated skin disease affecting at least 60 million people worldwide (1). Its most frequent form, plaque psoriasis, is characterized by the presence of salmon-pink cutaneous plaques covered by silvery scales in white skin and grey in black skin (2–5). The Psoriasis Area and Severity Index (PASI) is commonly used to evaluate disease severity and clinical outcome (6, 7). The vast majority of patients with psoriasis have mild-to-moderate disease, with topical therapy considered as the first-line treatment (8, 9). There is broad consensus that a mixed interleukin (IL)-17 (T helper type 17: Th17) and interferon gamma (IFNγ)/tumour necrosis factor (TNF) response (Th1/Tc1), driven by a vast network of cells, such as T and dendritic cells, plays a dominant role in promoting keratinocyte hyperproliferation and aberrant differentiation in psoriasis (10–12). Biologic agents targeting the IL-23/IL-17 immune axis and TNF signalling have revolutionized the treatment of moderate-to-severe psoriasis (11, 13, 14).

The role of CD8 T cells as significant sources of IL-17 (Type 17 CD8+T cells: Tc17) has also been extensively studied in the pathogenesis of psoriasis skin lesions (15–17). In psoriasis patients and mouse models, epidermal CD8 T cells exhibit an active Tc17 phenotype and blocking CD8 T cells prevents the development of psoriasis *in vivo* effectively (16, 18). Cheuk et al. reported that epidermal IL-17-producing CD8 T cells in psoriasis lesions co-express CD103 and are retained in resolved lesions after effective treatment, potentially contributing to local relapses (19). Additionally, circulating CD8 T cells predominantly respond with IFNγ production in patients with the HLA-Cw6 allele, which is associated with early-onset psoriasis (20–22). Beyond conventional CD8 T cell subsets, circulating mucosa-associated invariant T-cells (MAIT) also contribute to the pathogenesis of the disease (23–25).

Over the past decades, the association between systemic inflammation and local immune activation has received increased recognition (26–28), suggesting the contribution of circulating T cells to the pathogenesis of psoriasis (29). Although multiple investigations have explored the characteristics of peripheral blood CD4 T cell populations in individuals with psoriasis, examining numerical, phenotypic, and functional profiles, the findings have been inconsistent (30–33). Hence, the features and roles of circulating CD8 T cells in psoriasis patients are still ill-defined.

Dysregulation of skin immune homeostasis led by loss of immune tolerance has been frequently reported in psoriasis (34, 35). Hence, inhibitors of T cell co-stimulation have been investigated as novel therapeutic targets for disease control (36–38) and in experimental human and animal psoriasis models, as summarised by Yao and Liu et al. (39, 40). For instance, the immune checkpoint protein programmed death protein-1 (PD-1) was found to be expressed by cutaneous IL-17A-producing T cells in psoriasis patients and in psoriasis-like mouse models (41). Kim et al. also found that blockade of the PD-1 axis leads to suppressed anti-CD3-induced IL-17A production by γδ T cells in imiquimod (IMQ)-induced psoriasis-like skin inflammation (41). Additionally, Sgambelluri et al. reported a subset of blood CCR4/CD103-expressing CD8+ effector T cells significantly correlating with disease severity in patients with moderate-to-severe psoriasis (42). Therefore, delineating the roles of skin homing chemokines and co-signalling molecules on peripheral blood CD8+T cell compartments suspected to play a role in psoriasis becomes paramount.

In this study, we aimed to provide a deeper understanding of the phenotype, activation status, and function of circulating CD8 T cells and their potential implications in mild-to-moderate psoriasis. We used flow cytometry to characterise peripheral blood CD8 T cell populations, their frequency, expression of skin-trafficking chemokine receptors and co-signalling molecules compared to healthy subjects. We then assessed the functionality of circulating CD8 T cells by examining the expression of activation markers and the production of mediators, such as cytokines, upon activation. Multiparametric flow cytometry data was analysed using conventional manual gating and unsupervised clustering approaches.

## 2 Methods

### 2.1 Patients

This study was approved by the London Bridge Research Ethics Committee (REC number: 06/Q0704/18) and the University of Manchester (UREC ref: 2018-2696-5711). After obtaining informed consent, peripheral blood samples were collected from 7 psoriasis patients and 11 healthy subjects. Demographics and clinical characteristics of the enrolled subjects are summarised in **Supplementary Table 1** and **Supplementary Methods**.

### 2.2 PBMC isolation

Human peripheral blood mononuclear cells (PBMCs) were freshly isolated from whole blood using density gradient centrifugation (Ficoll-Paque) as previously described (43). Cell viability and concentration were determined using trypan blue staining and a counting chamber. Subsequently, PBMCs were frozen in a freezing solution containing fetal bovine serum (FBS) or human serum albumin supplemented with 10% Dimethyl Sulfoxide (DMSO) and stored at -80°C until use.

### 2.3 Multiparametric flow cytometry analysis and intracellular cytokine staining

Cryopreserved PBMCs were rapidly thawed and washed in pre-warmed RPMI medium (Sigma) supplemented with 10% FBS, 10% Penicillin-Streptomycin, and 2 mM L-glutamine. Thawed PBMCs were split across three staining panels for flow cytometry. For *ex vivo* immunophenotyping, chemokine and co-signalling molecule expression on CD8 T cells, PBMCs were plated in v-bottom 96-well plates (2-5 x 10^5^ cells/well). For *in vitro* stimulation, PBMCs were seeded in 96-well flat-bottom plates (2-5 x 10^5^ cells/well). The cells were subsequently subjected to two distinct activation protocols. One underwent stimulation with phorbol 12-myristate 13-acetate (PMA, 25 ng/ml) and ionomycin (ION, 710 ng/ml) (Merck) for 6 hours, targeting protein kinase C and calcineurin pathways. The second was exposed to plate-bound anti-CD3 (1 µg/ml) and soluble anti-CD28 (1 µg/ml) (Biolegend) for 24 hours. Both simulations were conducted in culture medium incubated at 37°C with 5% CO_2_. Unstimulated cells were included as negative controls. Brefeldin A (Sigma-Aldrich) was incorporated into the culture medium during the final 6 hours of stimulation to inhibit intracellular vesicular transport.

Cell surface and intracellular staining were conducted on *ex vivo*, unstimulated and stimulated PBMCs. Detailed information regarding fluorochrome-conjugated antibodies utilised is provided in **Supplementary Table 2**. For surface staining, PBMCs were initially blocked with Human True StainFcX (Biolegend) and subsequently stained for cell surface markers for 30 min at 4°C in the dark, followed by live/dead staining (Thermo Fisher Scientific). Subsequently, cells were fixed with 2% paraformaldehyde (PFA) for 10 min at room temperature (Thermo Fisher).

In samples designated for cytokine production detection, following surface and live/dead staining, cells were fixed and permeabilised using 1x permeabilization buffer (Invitrogen), then stained with antibodies against intracellular markers for 1 h at room temperature. After washing, cells were resuspended in FACS buffer (PBS, 2 mM EDTA, 2% FBS), and analysis was performed using an LSR Fortessa flow cytometer (BD Biosciences). The complete experimental design is delineated in **Supplementary Figure 1A**.

### 2.4 Flow cytometry data analysis

Flow cytometry files (FCS) were exported and compensated using single-stained UltraComp Beads (Invitrogen). The compensated data were then processed using FlowJo Software (BD Biosciences) to eliminate debris, non-viable cells, and cellular aggregates (**Supplementary Figure 1B**). This preparatory step preceded both manual and unsupervised clustering analyses.

Manual gating was performed on flow cytometry data using FlowJo. CD8+ T cell populations (CD3+CD56-CD8+ cells) were identified based on the gating strategy provided in **Supplementary Figure 1B** and **Supplementary Methods**. The expression levels of different markers were analysed and subsequently plotted using Prism 10 (GraphPad software).

Unsupervised analyses were carried out using OMIQ (Dotmatics). For a detailed description, see **Supplementary Methods**.

### 2.5 Statistical analysis

Data are presented as percentage of total populations, mean ± standard deviation (SD). In both manual gating and unbiased clustering, the Mann-Whitney U test was used to detect significant differences between groups in unpaired analyses. In unsupervised analyses, the raw p values were adjusted by the original Benjamini-Hochberg false discovery rate (FDR) method (44), using the *p.adjust* function in R (ver. 4.0.5), where applicable. EdgeR was used to screen FlowSOM-defined meta-clusters for significance based on cell counts in OMIQ and using a custom script in R for the analysis of unbiased clustering datasets (45). Spearman correlation and simple linear regression between PASI scores and identified subpopulations were calculated using Prism 10.3 (Graphpad). Adjusted p values < 0.05 were considered significant for all the analyses.

## 3 Results

### 3.1 Immuno-phenotyping reveals normal distribution of CD8 T cells, NK and NKT cells but reduced frequencies of CD8 memory T cells in psoriasis

Several studies have quantified the composition of immune cell populations in the peripheral blood of psoriasis patients, primarily focusing on those with moderate-to-severe phenotype (46–49). However, limited data exist on the distribution of CD8 T cell subsets in patients with mild-to-moderate psoriasis. To explore this, PBMCs were stained with a 14-color antibody panel to characterise lymphocytes and specific CD8 T cell subsets (**Supplementary Table 2**).

**Figure 1:**
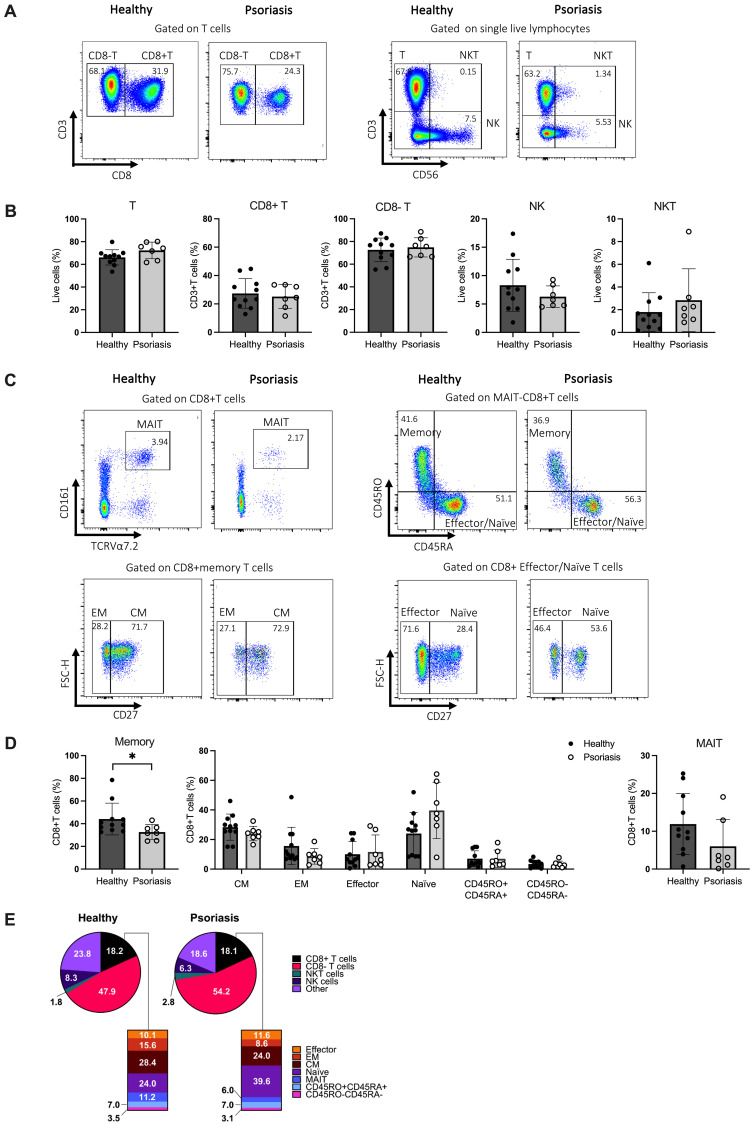
CD8+ immune cell subpopulations in the blood of patients with mild-to-moderate psoriasis are comparable to those of healthy individuals. Peripheral blood mononuclear cells (PBMCs) from mild-to-moderate psoriasis patients (n=11) and healthy individuals (n=7) were labelled with different cell surface and chemokine markers and analysed using flow cytometry. **(A)** Representative plots of the chosen gating strategy. **(B)** Frequency of identified T, CD8+ T, CD8- T, NK, and NKT immune cell subsets in healthy and psoriasis subjects. **(C)** Representative plots of the chosen gating strategy **(D)** Frequency of CD8+ T cell subpopulations in mild-to-moderate psoriasis and healthy individuals. **(E)** Pie and bar charts of summarised results. Significant differences were analysed using the Mann-Whitney U test. *p* value < 0.05. Bar plots represent mean value ± SD. CM, central memory T cells; EM, effector memory T cells; Effector, effector T cells; MAIT, mucosal-associated invariant T cells; Naïve, naïve T cells; NK, natural killer cells; NKT, natural killer T cells; T, T cells.

As shown in **Figures 1A, B**, the frequencies of total T cells, CD8^-^T cells, natural killer (NK) and NKT cells were comparable between psoriasis patients and healthy controls, in line with previous reports (62–64). The overall frequency of CD8 T cells in psoriasis patients showed considerable variability but was also similar to healthy controls. We then investigated the distribution of CD8 T cell subsets within the total population by examining lineage cell surface markers, as summarised in **Supplementary Table 3**. The frequency of CD8 memory T cells was significantly decreased in psoriasis patients compared to healthy controls (*p*<0.05). However, we did not observe significant differences in the frequencies of central memory (CM) (CD45RA-CD45RO+CD27+), effector memory (EM) (CD45RA-CD45RO+CD27-), naïve (CD45RA+CD45RO-CD27+) and effector (CD45RA+CD45RO-CD27-) CD8 T cells, nor MAIT cells (TCRVα7.2+CD161+) between psoriasis patients and healthy controls (**Figures 1C, D**). A summary of the analysed immune cell and T cell subsets is presented in **Figure 1E** and **Supplementary Table 4**. No significant differences were found in the CD8+/CD8- ratio (data not shown).

In conclusion, these results indicate a normal distribution of circulating CD8 T cells in mild-to-moderate psoriasis.

### 3.2 Circulating CD8 T cells and MAIT CD8 cells from psoriasis patients display reduced CXCR3 and increased CCR4 expression compared to healthy controls

There is accumulating evidence that circulating memory T cells are associated with cutaneous manifestations in psoriasis (50). Since our data indicate a reduction in memory CD8 T cell frequency in psoriasis patients, we have next investigated the CD8 T cell phenotype, particularly the expression of receptors associated with tissue homing/trafficking chemokines.

As shown in **Supplementary Figure 2**, we observed no significant difference in CD69, CD103, CCR4 and CLA expression on CD8 T cells between psoriasis patients and healthy controls. However, CCR4 was expressed at a higher frequency in the CD8 MAIT cell compartment in psoriasis than in healthy subjects (**Supplementary Figure 2B**). CXCR3 expression was, instead, reduced in CD8 and CD8 memory T cell compartments in psoriasis compared to healthy controls (**Supplementary Figure 2F**). When analysing different CD8 T cell subpopulations, CXCR3 was particularly reduced in CM CD8 T cells. Conversely, the expression of the other homing/trafficking markers CD69, CD103, CLA and CCR4 was low across all CD8 T cell subsets in both cohorts studied (**Supplementary Figure 3**). Beyond CD8 T cells, the expression of CCR4, CD103 and CLA was significantly decreased in CD8-T cells, CXCR3 in NK cells and CD69 and CLA in NKT cells (**Supplementary Figures 2B–E**). However, unsupervised OMIQ analyses did not replicate these findings (see **Supplementary Results**).

In conclusion, these results hint at possible differences in the expression of receptors associated with tissue homing in CD8 T cells, namely CXCR3 and CCR4, from patients with mild-to-moderate psoriasis compared to healthy controls.

### 3.3 BTLA expression is increased in CD8 effector T cells

Co-signalling receptors play a role in modulating T cell activities. The expression of T-cell immunoreceptor with immunoglobulin andimmunoreceptor tyrosine-based inhibitory motif domains (TIGIT) on circulating CD4 T cells correlates with psoriasis severity (33, 51). Thus, to further dissect the phenotype of circulating CD8 T cells in psoriasis patients with mild-to-moderate disease severity, we examined the expression of co-signalling molecules (co-stimulatory: CD28, CD134 (OX40L), LIGHT; co-inhibitory: TIGIT, programmed cell death protein 1 (PD-1), BTLA) on circulating CD8 T cells.

**Figure 2:**
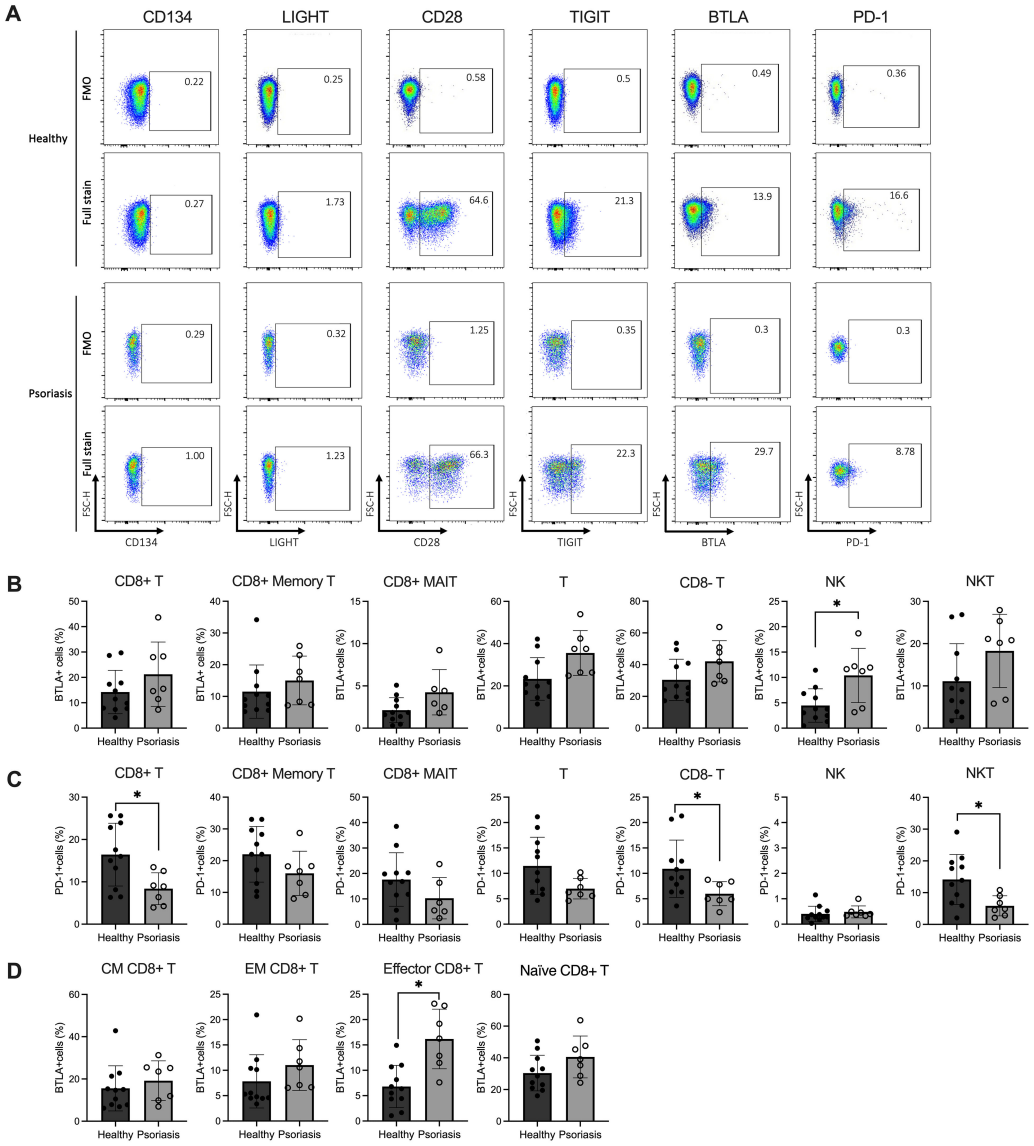
Expression levels of BTLA and PD-1 are variable in circulating lymphocyte subsets of patients with mild-to-moderate psoriasis. Peripheral blood mononuclear cells (PBMCs) were isolated from mild-to-moderate psoriasis patients (n=11) and healthy individuals (n=7) and analysed in flow cytometry. **(A)** Representative flow plots of co-stimulatory/inhibitory marker expression on CD8 T cells. Bar plots show the relative percentages (y-axis) of **(B)** BTLA+ and **(C)** PD-1+ CD8 T cells and other immune cell subsets, and **(D)** BTLA expression measured on circulating CD8 T cell subpopulations (CM, EM, Eff and Naïve) in psoriasis compared to healthy individuals. Differences were calculated using the Mann-Whitney U test. *p value < 0.05. Bar plots represent mean value ± SD. CM, central memory T cells; EM, effector memory T cells; Eff, effector T cells; MAIT, mucosal-associated invariant T cells; Naïve, naïve T cells; NK, natural killer cells; NKT, natural killer T cells; T, T cells.

As shown in **Figure 2A**, while CD28, TIGIT, BTLA and PD-1 are expressed by CD8 T cells, the costimulatory receptors LIGHT and CD134 are minimally expressed in both psoriasis patients and healthy controls (<2%). Although there was no difference in the expression of BTLA by CD8 T cells between the two cohorts (**Figure 2B**), upon examining CD8 T subsets, we found significant BTLA upregulation in CD8 effector T cells in psoriasis (*p*<0.005) (**Figure 2D**). BTLA expression was also increased in NK cells from psoriasis patients compared to healthy controls (*p*<0.05, **Figure 2B**).

As for the remaining markers, the expression of CD28 and TIGIT was similar between CD8 T cells and other cell subtypes in both healthy and psoriasis, similar to a previous report (52)(**Supplementary Figure 4**). Conversely, PD-1 expression was significantly decreased in CD8 T cells, CD8- T cells and NKT cells compared to healthy controls (**Figure 2C**). When performing unsupervised analyses, however, we could not find significant changes in costimulatory/co-inhibitory marker frequencies in CD8 T cell sub-populations. Still, we identified differences in NKT cell subset frequencies between healthy and psoriasis patients, further discussed in **Supplementary Results**.

In summary, our data demonstrate increased BTLA expression in CD8 effector T cells and reduced PD-1 expression in all CD3-expressing subpopulations of patients with mild-to-moderate psoriasis, suggesting possible variation in T cell function if these co-inhibitory receptors are engaged.

### 3.4 Circulating CD8 T cells are functionally impaired in patients with psoriasis

It is widely recognised that the increased release of pro-inflammatory cytokines, such as IL-23, IL-17 and TNF, plays a critical role in developing psoriasis (53). To further investigate the changes in the effector function of circulating CD8 T cells in psoriasis, two *in vitro* stimulation methods, TCR-dependent (anti-CD3/CD28) and -independent (PMA/ION), were employed to assess the production of cytokines by T cells *in vitro*, as this varies significantly depending on the type of stimulus used (54–56). Following stimulation, the activation status (CD69) and the expression of mediators (Granzyme B, TNF, IFNγ and IL-17A/F) were assessed by surface and intracellular staining using flow cytometry.

**Figure 3:**
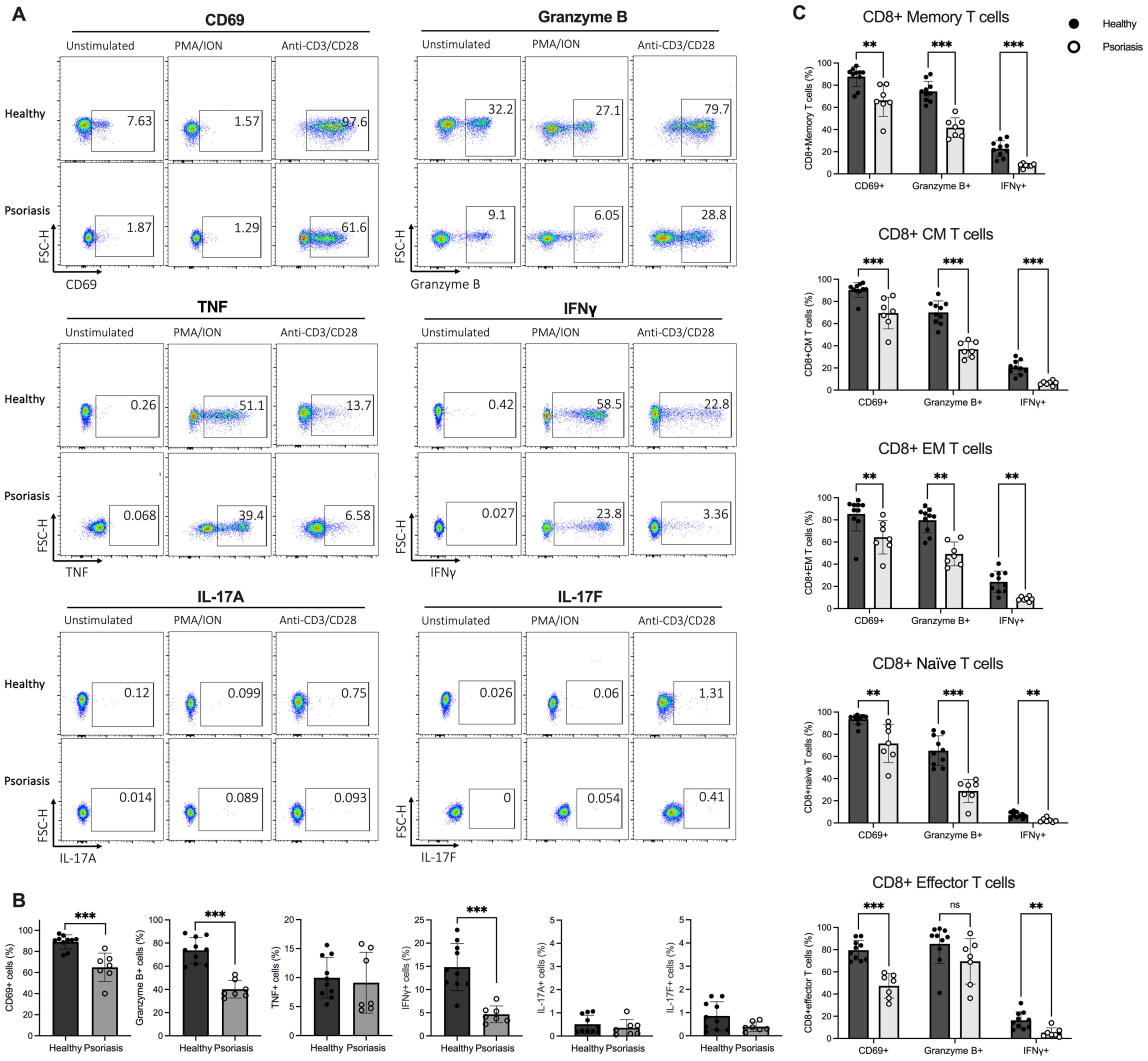
Stimulated CD8 T cells from psoriasis patients display reduced CD69, Granzyme B and IFNγ expression compared to healthy individuals. **(A)** Peripheral blood mononuclear cells (PBMCs) isolated from psoriasis patients (n=7) and healthy controls (n=10) were stimulated with anti-CD3/CD28 or phorbol 12-myristate 13-acetate/ionomycin (PMA/ION) and membrane and intracellular expression of mediators was analysed in flow cytometry. Representative flow plots showing expression of CD69, Granzyme B, TNF, IFNγ, IL-17A and IL-17F on CD8 T cells. **(B)** Bar plots show the percentage of CD69+, Granzyme B+, TNF+, IFNγ+, IL-17A+ or IL-17F+ CD8 T cells when stimulated with antiCD3/CD28. **(C)** Bar plots show the percentage of CD69+, Granzyme B+ and IFNγ+ cells within CM, EM, Eff and Naïve CD8 T cell subsets in psoriasis compared to healthy controls when stimulated with anti-CD3/CD28. Data are shown as mean ± SD. Differences between groups were calculated using the Mann-Whitney U test. **p < 0.005, ***p < 0.0005. CM, central memory T cells; EM, effector memory T cells; Effector, effector T cells; Naïve, naïve T cells; ns, not significant.

As shown in the representative flow plots in **Figure 3A**, incubation with PMA/ION significantly increased the intracellular expression of IFNγ compared to anti-CD3/CD28 stimulation (*p*<0.005). In contrast, anti-CD3/CD28 exposure preferentially upregulated intracellular Granzyme B and membrane CD69 expression. IL-17A and IL-17F production by CD8 T cells was not significantly affected by either stimulation method.

The frequency of CD69+, Granzyme B+ and IFNγ+ CD8 T cells in psoriasis patients was significantly lower following anti-CD3/CD28 stimulation (*p*<0.0005) (**Figure 3B**), suggesting downregulated TCR-dependent activation and impaired Tc1 cytokine production in CD8 T cells from psoriasis patients. Conversely, no significant difference in mediator expression was detected between psoriasis patients and healthy controls when CD8 T cells were stimulated in a TCR-independent manner (PMA/ION) (**Supplementary Figure 5**).

Differences in CD69, Granzyme B and IFNγ expression were also assessed in CD8 T cell subsets. Total memory, central memory, effector memory, and naïve CD8 T cell subpopulations from psoriasis patients exhibited a significantly lower percentage of CD69+, Granzyme B+, and IFNγ+ cells compared to healthy controls when stimulated with anti-CD3/CD28. Similarly, effector CD8 T cells showed a significantly lower CD69 and IFNγ expression in psoriasis patients. However, Granzyme B expression was not statistically different (**Figure 3C**). These results indicate that circulating CD8 T cells globally are functionally impaired in patients with mild-to-moderate psoriasis.

**Figure 4:**
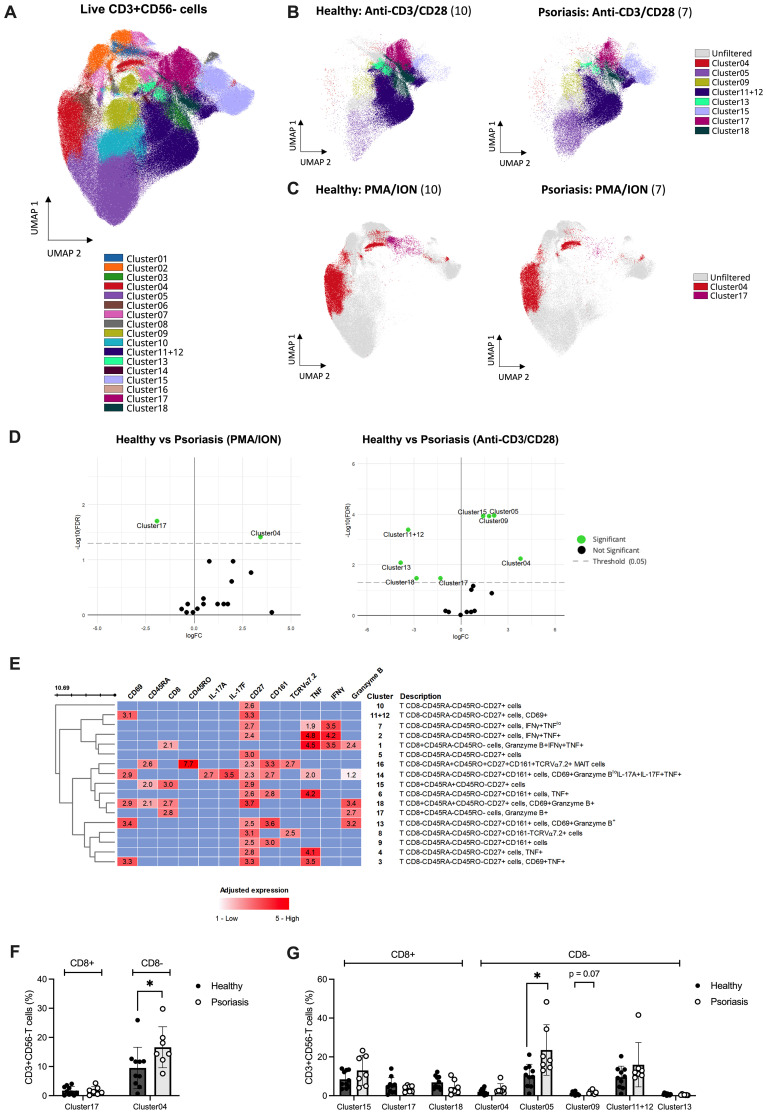
Unsupervised analysis reveals differences in mediator expression profile of T cell subsets following *in vitro* stimulation between psoriasis and healthy subjects. Peripheral blood mononuclear cells (PBMCs) isolated from psoriasis patients (n=7) and healthy controls (n=10) were stimulated with anti-CD3/CD28, phorbol 12-myristate 13-acetate/ionomycin (PMA/ION) or left unstimulated. Membrane and intracellular expression of mediators within manually gated CD3+CD56- T cells were analysed using OMIQ. **(A)** 18 identified FlowSOM clusters were projected onto two UMAP dimensions. The overlay plot shows concatenated event from all patient and healthy samples analysed (unstimulated, PMA/ION and anti-CD3/CD28) **(B)** from anti-CD3/CD28 or **(C)** PMA/ION stimulated PBMCs from psoriasis patients and healthy controls. Significant clusters identified using EdgeR (coloured) are overlayed onto non-significant clusters (gray) in **(B–D)** Volcano plot displaying the differential count abundance of identified clusters between psoriasis and healthy subjects calculated using EdgeR and plotted using EnhancedVolcano. **(E)** Heatmap of cell clusters identified by FlowSOM. Rows represent each identified cluster with a description of the phenotype on the right, and columns represent the markers of interest. Adjusted expression values were defined as higher than a specified threshold based on concatenated FMO controls for each marker in flow cytometry. **(F)** Manual validation of found significant clusters in PMA/ION and **(G)** anti-CD3/CD28 stimulations. Graphs represent mean ± SD. Differences between groups were calculated using Mann-Whitney U. *p value < 0.05. FC, fold change; FDR, false discovery rate; FMO, fluorescence minus one; UMAP, uniform manifold approximation and projection.

Finally, to investigate the differences in T cell activation and mediator expression using a more holistic approach, we analysed the entire dataset using OMIQ, selectively focusing on the CD3+CD56- cell population (**Figures 4A–E**). A total of 18 different meta-clusters, 4 of which CD8+ and 14 CD8- T cells, were identified (**Figure 4E**). Based on EdgeR analysis, three CD8 T cell clusters exhibited significant alterations in their count abundance across stimulations when comparing psoriasis patients with healthy individuals (**Figures 4B–E**).

Upon anti-CD3/CD28 stimulation, CD8+CD45RA+CD45RO-CD27+ T cells (Cluster 15) were significantly increased in psoriasis compared to the healthy cohort. Instead, CD8+CD45RA-CD45RO- T cells expressing Granzyme B (Cluster 17) and CD8+CD45RA+CD45RO-CD27+ naïve T cells expressing CD69 and Granzyme B (Cluster 18) were significantly downregulated (**Figure 4D**, right). Similarly, upon PMA/ION stimulation, CD8+CD45RA-CD45RO- T cells expressing Granzyme B (Cluster 17) were downregulated in psoriasis patients compared to healthy volunteers (**Figure 4D**, left). Of the identified CD8+ clusters using EdgeR, only Cluster 15 (including CD8+CD45RA+CD45RO-CD27+ T cells) was found significantly increased when analysing its frequency relative to total CD3+CD56- cells (FDR-adjusted p=0.016, **Supplementary Table 5**), but was not confirmed by manual gating analysis (**Figures F, G**). CD8- populations found differentially expressed using OMIQ are summarised in **Supplementary Table 5** and **Supplementary Results**.

In summary, supervised and unsupervised analyses of circulating CD8 T cell subsets demonstrate reduced TCR-dependent functional responses in patients with mild-to-moderate psoriasis compared to healthy controls.

### 3.5 IFNγ-producing CD8 T memory subsets correlate with psoriasis severity

To define the impact of disease severity on T cell subpopulations, we performed correlation and linear regression analysis between PASI scores and frequencies of found T cell subsets using supervised and unsupervised approaches.

Despite only in unadjusted analyses, we found that frequencies of CD45RA+CD45RO+ and IFNγ-producing memory CD8 T cells based on manual gating positively correlated with PASI scores. Conversely, CXCR3+ MAIT CD8- cells negatively correlated with disease severity (**Table 1**).

**Table 1:** Correlation between psoriasis area and severity index (PASI) scores and manually-gated CD8 T cell subset frequencies in psoriasis patients (n=7).

Condition	Description	r	p value	FDR-adjusted p value	R^2^	p value LR
Ex vivo	T CD8+CD45RA+CD45RO+ cells	0.8108	**0.0349**	0.3746	0.4833	0.0829
Ex vivo	CD8-CXCR3+CD161+TCRVα7.2+ MAIT cells	-0.8789	**0.0167**	0.3500	0.8532	**0.0030**
PMA/ION	T CD8+CD45RA-IFNγ+ Memory cells	0.8727	**0.0167**	0.5208	0.5170	0.0686
PMA/ION	T CD8+CD45RA-CD27-IFNγ+ EM cells	0.8469	**0.0238**	0.5208	0.7389	**0.0131**

In bold p values < 0.05. EM, effector memory; FDR, false discovery rate; IFNg, interferon gamma; LR, linear regression; MAIT, mucosal-associated invariant T cells; r, Spearman correlation.

In unsupervised analyses, we found significant negative correlations between PASI scores and CD8- T subsets in both ex vivo and stimulated conditions, summarised in **Supplementary Results** and **Supplementary Table 6**.

These results suggest that, in mild-to-moderate psoriasis, memory CD8 T cells, in particular IFNγ-producing EM populations, and CD8- T cells, particularly TNF-producing cells, are modulated in function of the disease severity.

## 4 Discussion

This study characterised the peripheral blood CD8 T compartment in psoriasis patients using both manual and unsupervised analytical tools (57). While many published studies on CD8 T cells in psoriasis have primarily focused on moderate-to-severe cases (16, 42, 46–48, 58, 59), our research selectively examined the immunological features of subjects with mild-to-moderate psoriasis. This group represents more than 80% of individuals affected by the condition and is associated with lower odds of comorbidities compared to those with severe disease (60). Our findings demonstrate a normal distribution of circulating CD8 T cells with a significant decrease in CD8 memory T cells, higher expression of BTLA in CD8 effector T cells, and an impaired immune response in circulating CD8 T cell subsets.

Zecevic-Pasic et al. reported a negative correlation between NK lymphocyte numbers and PASI while T cells (including CD4+ and CD8+ subsets) showed no significant difference between psoriasis and control cohorts (61). Furthermore, significant differences in circulating T cells have been documented between mild and more severe forms of psoriasis, using a PASI score of 12 as a cut-off (62). Our study shows that patients with mild-to-moderate psoriasis have normal frequencies of circulating CD3, CD8, NK and NKT cells compared to controls. While this finding is consistent with published studies that investigate cohorts including patients with mixed disease severity, discrepancies remain with studies focusing on severe disease cohorts (62–64).

In our study, naïve T cells were found to be comparable between the two cohorts studied, yet highly variable among donors. Demographic factors, such as age, have been reported to influence lymphocyte subset frequency. This may help explain the increased variation in cell subset frequencies observed across different studies (65–67). Notably, our findings indicate that psoriasis patients have significantly decreased circulating memory CD8 T cells, particularly CCR7- EM phenotype (gating strategy refers to **Supplementary Figure 1**), compared to healthy controls, and cell subsets with effector functions expressing Granzyme B (68, 69). In contrast, Langewouters et al. demonstrated comparable levels of CD8+CD45RO+ T cells between patients with PASI<12 and healthy individuals (62). The discrepancy between the current literature and our findings could stem from three factors. First, our study used double CD45RO and CD45RA labelling to identify memory or naïve T cell populations. Gating strategies using two different T cell subset markers, CD27 or CCR7, produced divergent results in identifying CD8 T cell sub-populations (**Supplementary Figure 6**), and this outcome aligns with previous research (66, 70, 71). Second, the definition of mild-to-moderate disease varies significantly between investigators. Currently, the “rule of 10” applies to PASI scores, which dictates access to biological treatment (72). However, in contrast to our study, some authors selected an arbitrary cut-off to include subjects with borderline scores (i.e., between 10 and 12) in the definition of moderate disease (33, 61, 62, 73, 74). Third, it remains uncertain whether the reduction in CD8+ memory T cells in the peripheral blood is due to the migration of these cells into skin lesions during an active phase of the disease. However, this would support the findings that CD8 T cells and memory T cells are increased in spreading psoriatic plaques (75).

CD8 T cell subsets at various stages of cell differentiation exhibit selective/shifted expression of CCR4 and other skin-tropic chemokines associated with systemic and/or cutaneous inflammation in psoriasis (42, 50, 76, 77). While a positive correlation between PASI and circulating CLA+ T cells in psoriasis patients has been previously observed, another study showed no difference in CLA expression in either circulating CD8 or CD4 T cells between patient and healthy cohorts (78–80). In our study, we could not show significant differences in the expression of CLA in CD8 T cells in mild-to-moderate psoriasis patients compared to healthy controls. However, our cohort did not discontinue topical treatment before sample collection, and we cannot exclude the possibility that treatment may have affected the expression of tissue-homing receptors such as CLA. Furthermore, Sigmundsdóttir et al. reported that systemic treatment can significantly influence the expression of the skin tropic marker CLA on blood T cells (80, 81)

We have investigated the expression of co-inhibitory and co-stimulatory receptors on circulating CD8 T cells of our target population. Our findings did not reveal any major dysregulation. However, we did observe a significant increase in BTLA expression on CD8 effector T cells. This possibly suggests suppressing functions of CD8 effector T cells in patients with mild-to-moderate psoriasis. Our results align with previous publications, pointing at no significant dysregulation of co-inhibitory BTLA on total CD8 T cells in mild psoriasis patients (PASI<10).

Several studies have investigated CD8 T cell function in psoriasis (18, 24, 82). Bose and colleagues demonstrated that patients with psoriasis exhibited no significant difference in cytokine production, including IFNγ, TNF, and IL-2, compared to healthy donors using anti-CD3-activated PBMCs (83, 84). A recent study phenotyping circulating CD8 T cells revealed comparable production of selected cytokines in CD8 MAIT and recirculating memory CD8 T cell clusters (32). In our study, we applied two *in vitro* stimulation methods with different activation mechanisms (TCR-dependent and -independent). This allowed us to broadly assess the functionality of circulating CD8 T cells, specifically the production of pro-inflammatory mediators, to elucidate their contribution to the pathogenesis of psoriasis. We observed reduced functionality of peripheral blood CD8 T cells from psoriasis patients compared to healthy control, with a significant decrease in CD69, Granzyme B and IFNγ expression across CD8 T cell subsets following TCR-dependent stimulation.

IL-17 has been considered as a hallmark cytokine of psoriasis pathogenesis (73). We studied circulating CD8 T cells producing IL-17A or IL-17F and observed low and comparable levels of IL-17A/F expression in both patient and control cohorts upon *in vitro* stimulation. This could be due to differences in stimulation protocols, as discussed by Manescu et al. (85). Previous studies have shown that activated CD4 T cells, rather than CD8 T cells, are considered the main source of IL-17 in the blood (84, 86) Significantly decreased Th17 cells and IL-17 expression were also found in peripheral blood and skin of psoriasis patients treated with topical therapy (87, 88). In an imiquimod-induced psoriasiform mouse model, treatment with topical steroids significantly decreased the levels of inflammatory cytokines TNF, IL-23 and IL-17 in psoriatic lesions (89).

IFNγ plays a significant role in the pathogenesis of psoriasis, as it is often found elevated in both serum and lesional skin of affected patients (90–93). Our results indicate a significant correlation between IFNγ-expressing, PMA/ION-activated CD8 memory T cell populations and PASI scores. This aligns with previous studies conducted on peripheral blood, proposing IFNγ as a biomarker to monitor disease progression (94), and response to successful anti-TNF treatment on skin transcriptomics data (93). In psoriatic skin lesions, tissue-resident CD8 T memory cells possess higher IFNγ-producing capacity than CD4 T cells (19, 95). However, further research is necessary to elucidate whether IFNγ-producing CD8 T memory cells in lesional skin result from the migration of circulating memory pools and activation *in situ* in patients with psoriasis.

We also found a positive correlation between double-positive CD45RO+CD45RA+ CD8 T cell frequency and disease activity. Prince et al. characterised circulating double-positive T cells as having an intermediate activation status (96). There is currently limited information on double-positive T cell subsets in the context of psoriasis. Cytometry by time-of-flight analysis of PBMCs from psoriasis patients and healthy controls showed comparable frequency of terminally differentiated effector memory (TEMRA) CD8 T cells, which are a subset of CD45RO-CD45RA double-positive T cells lacking CD27 expression (49). Interestingly, a recent paper investigating synovial tissues in patients with psoriatic arthritis found that T cell clusters co-expressing CD45RO and CD45RA show extensive interaction with vascular cells, macrophages, DCs and other T cells (97).

We also found that circulating CXCR3+CD8- MAIT cells in psoriasis negatively correlate with PASI scores, hinting at the possible recruitment of CD8- MAIT cells expressing the trafficking receptor CXCR3 from the circulation to inflamed skin in mild disease. As MAIT cells display an EM-like phenotype (98), a similar correlation between circulating CXCR3+CD4+ EM T cells and PASI scores was observed in patients with psoriasis (50).

While manual analysis is the current standard for analysing flow cytometry data, increasingly more applications of unsupervised clustering algorithms have been documented, paralleling the analyses done in mass cytometry (30, 32, 99–101). Unsupervised analyses present the advantage of being user-independent and capable of identifying minute populations that could be missed when analysing sets manually. Several pipelines have been proposed, differing in scaling and/or normalisation techniques, inclusion of FMO controls and clustering methods (102–104). However, some pitfalls are unique to this technique and especially relevant in the context of flow cytometry-based analyses. First, including all markers in an effort to capture populations of interest can lead to identifying multiple clusters with similar characteristics, a process known as over-clustering. This situation often necessitates manual intervention, as discussed by Baumgaertner et al. (57). Second, unlike mass cytometry datasets, flow cytometry data present unique challenges, such as donor autofluorescence, which complicates pooling samples together, as donor-dependent anomalies can produce clustering on outliers (105). In light of the methodological differences between the two strategies, we combined both manual and unsupervised analyses, offering a more comprehensive view of the results obtained.

## 5 Limitations

This study presents experimental design and analytical limitations worth addressing. The sample size, particularly of psoriasis patients (n=7), was small and unequal to healthy subjects (n=11), with limited power to discriminate minute changes in cell subset frequencies or receptor expression.

All chosen patients underwent topical treatment with either steroids or calcipotriol at the time of sample collection. This could have contributed to the reduced activation observed compared to healthy subjects in functional assays and the lower representation of re-circulating cellular pools from the skin to the blood in psoriasis patients, as previously argued.

Other than PASI scores and treatment, clinical features, including HLA-Cw6 and HLA-DRB1*07 haplotype determination, were unavailable. These features could have helped determine the susceptibility to severe disease (20, 106) and further identify potential CD8 T subsets of interest. Similarly, our study only included Caucasian subjects, limiting the translation of the results to other ethnic groups.

In our study, we performed supervised and unsupervised analyses to capture different aspects of the same dataset. However, the mutual interpretability of manual and unsupervised analyses was limited by several factors. First, the unsupervised analyses were performed using arcsinh-transformed and batch-corrected files, which differed from the original files. Second, in some unsupervised analyses, we found markers below the positivity threshold when adjusting expression to FMO controls, which contrasted with manual analyses. We speculate that high donor-specific autofluorescence affects the possibility of investigating populations with very low marker expression. This is especially relevant when adjusting the expression to pooled fluorescence controls, as shifts in fluorescence from subjects with high backgrounds can mask donors with lower backgrounds.

Thus, discrepancies in the functional profiles of CD8 T cells compared to available studies can be attributed to multiple causes, including variations in experimental settings, data analysis techniques, size of the patient cohorts, and treatment, highlighting the need for further investigations.

## 6 Conclusion

Our findings highlight abnormalities in the phenotype and functionality of circulating CD8 T cells in patients with mild-to-moderate psoriasis. Whether the suppressed expression of CD8 T cell mediators contributes to the pathogenesis of mild-to-moderate psoriasis or results from topical treatment remains to be determined. Our study emphasises the need for a deeper understanding of CD8 T cell subsets in relation to psoriasis severity and treatment. Expanding the antibody panel to include markers for phenotype, skin homing chemokines, co-signalling molecules, and cytokines could more accurately identify relevant CD8 T cell subtypes. Furthermore, improved disease stratification within the patient cohort, considering demographic and clinical variables, could provide a clearer understanding of CD8 T cell alterations and their correlation with disease outcomes, potentially aiding therapy optimisation (107, 108).

## Data Availability

The raw data supporting the conclusions of this article will be made available by the authors, without undue reservation.

## References

[1] Parisi R Iskandar IY Kontopantelis E Augustin M Griffiths CE Ashcroft DM. National, regional, and worldwide epidemiology of psoriasis: systematic analysis and modelling study. bmj. (2020) 369:m1590. doi: 10.1136/bmj.m1590 32467098 PMC7254147

[2] Albanesi C. Immunology of psoriasis. In: Clinical immunology. Netherlands: Elsevier (2019). p. 871–878.e1.

[3] Griffiths CE Barker JN. Pathogenesis and clinical features of psoriasis. Lancet. (2007) 370:263–71. doi: 10.1016/S0140-6736(07)61128-3 17658397

[4] Armstrong AW Read C. Pathophysiology, clinical presentation, and treatment of psoriasis: a review. Jama. (2020) 323:1945–60. doi: 10.1001/jama.2020.4006 32427307

[5] Bowcock AM Cookson WO. The genetics of psoriasis, psoriatic arthritis and atopic dermatitis. Hum Mol Genet. (2004) 13:R43–55. doi: 10.1093/hmg/ddh094 14996755

[6] Ashcroft D Li Wan Po A Williams H Griffiths C. Clinical measures of disease severity and outcome in psoriasis: a critical appraisal of their quality. Br J Dermatol. (1999) 141:185–91. doi: 10.1046/j.1365-2133.1999.02963.x 10468786

[7] Augustin M Gottlieb AB Lebwohl M Pinter A Warren RB Puig L. Complete skin clearance is associated with the greatest benefits to health-related quality of life and perceived symptoms for patients with psoriasis. Dermatol Ther. (2024) p:1–17. doi: 10.1007/s13555-024-01261-6 PMC11480266 39285121

[8] Kim WB Jerome D Yeung J. Diagnosis and management of psoriasis. Can Family Physician. (2017) 63:278–85. PMC5389757 28404701

[9] Papp KA Gniadecki R Beecker J Dutz J Gooderham MJ Hong C-H. Psoriasis prevalence and severity by expert elicitation. Dermatol Ther. (2021) 11:1053–64. doi: 10.1007/s13555-021-00518-8 PMC8163919 33886086

[10] Lowes MA Kikuchi T Fuentes-Duculan J Cardinale I Zaba LC Haider AS. Psoriasis vulgaris lesions contain discrete populations of Th1 and Th17 T cells. J Invest Dermatol. (2008) 128:1207–11. doi: 10.1038/sj.jid.5701213 18200064

[11] de Alcantara CC Reiche EMV Simão ANC. Cytokines in psoriasis. Adv Clin Chem. (2021) 100:171–204. doi: 10.1016/bs.acc.2020.04.004 33453865

[12] Fitch E Harper E Skorcheva I Kurtz SE Blauvelt A. Pathophysiology of psoriasis: recent advances on IL-23 and Th17 cytokines. Curr Rheumatol Rep. (2007) 9:461–7. doi: 10.1007/s11926-007-0075-1 PMC2893221 18177599

[13] Veale DJ Fearon U. The pathogenesis of psoriatic arthritis. Lancet. (2018) 391:2273–84. doi: 10.1016/S0140-6736(18)30830-4 29893226

[14] Nash P Kirkham B Okada M Rahman P Combe B Burmester G-R. Ixekizumab for the treatment of patients with active psoriatic arthritis and an inadequate response to tumour necrosis factor inhibitors: results from the 24-week randomised, double-blind, placebo-controlled period of the SPIRIT-P2 phase 3 trial. Lancet. (2017) 389:2317–27. doi: 10.1016/S0140-6736(17)31429-0 28551073

[15] Kim J Moreno A Krueger JG. The imbalance between Type 17 T-cells and regulatory immune cell subsets in psoriasis vulgaris. Front Immunol. (2022) 13:1005115. doi: 10.3389/fimmu.2022.1005115 36110854 PMC9468415

[16] Piskin G de Boer OJ van der Loos CM Teeling P Bos JD Teunissen MB. Overrepresentation of IL-17A and IL-22 producing CD8 T cells in lesional skin suggests their involvement in the pathogenesis of psoriasis. PloS One. (2010) 5:e14108. doi: 10.1371/journal.pone.0014108 21124836 PMC2991333

[17] Hijnen D Knol EF Gent YY Giovannone B Beijn SJ Kupper TS. CD8+ T cells in the lesional skin of atopic dermatitis and psoriasis patients are an important source of IFN-γ, IL-13, IL-17, and IL-22. J Invest Dermatol. (2013) 133:973–9. doi: 10.1038/jid.2012.456 PMC3835628 23223131

[18] Di Meglio P Villanova F Navarini AA Mylonas A Tosi I Nestle FO. Targeting CD8+ T cells prevents psoriasis development. J Allergy Clin Immunol. (2016) 138:274–276.e6. doi: 10.1016/j.jaci.2015.10.046 26782974

[19] Cheuk S Wikén M Blomqvist L Nylén S Talme T Ståhle M. Epidermal Th22 and Tc17 cells form a localized disease memory in clinically healed psoriasis. J Immunol. (2014) 192:3111–20. doi: 10.4049/jimmunol.1302313 PMC3962894 24610014

[20] Chen L Tsai TF. HLA-cw6 and psoriasis. Br J Dermatol. (2018) 178:854–62. doi: 10.1111/bjd.16083 29072309

[21] Ikäheimo I Tiilikainen A Karvonen J Silvennoinen-Kassinen S. HLA risk haplotype Cw6, DR7, DQA1* 0201 and HLA-Cw6 with reference to the clinical picture of psoriasis vulgaris. Arch Dermatol Res. (1996) 288:363–5. doi: 10.1007/BF02507104 8818183

[22] Johnston A Gudjonsson J Sigmundsdottir H Love T Valdimarsson H. Peripheral blood T cell responses to keratin peptides that share sequences with streptococcal M proteins are largely restricted to skin-homing CD8+ T cells. Clin Exp Immunol. (2004) 138:83–93. doi: 10.1111/j.1365-2249.2004.00600.x 15373909 PMC1809187

[23] Raychaudhuri SK Abria C Mitra A Raychaudhuri SP. Functional significance of MAIT cells in psoriatic arthritis. Cytokine. (2020) 125:154855. doi: 10.1016/j.cyto.2019.154855 31541902

[24] Volarić I Vičić M Prpic-Massari L. The role of CD8+ T-cells and their cytokines in the pathogenesis of psoriasis. Acta Dermatovenerologica Croatica. (2019) 27:159–9. 31542059

[25] Leijten EF van Kempen TS Olde Nordkamp MA Pouw JN Kleinrensink NJ Vincken NL. Tissue-resident memory CD8+ T cells from skin differentiate psoriatic arthritis from psoriasis. Arthritis Rheumatol. (2021) 73:1220–32. doi: 10.1002/art.41652 PMC8362143 33452865

[26] Korman N. Management of psoriasis as a systemic disease: what is the evidence? Br J Dermatol. (2020) 182:840–8. doi: 10.1111/bjd.18245 PMC7187293 31225638

[27] Grozdev I Korman N Tsankov N. Psoriasis as a systemic disease. Clinics Dermatol. (2014) 32:343–50. doi: 10.1016/j.clindermatol.2013.11.001 24767182

[28] Campanati A Marani A Martina E Diotallevi F Radi G Offidani A. Psoriasis as an immune-mediated and inflammatory systemic disease: from pathophysiology to novel therapeutic approaches. Biomedicines. (2021) 9:1511. doi: 10.3390/biomedicines9111511 34829740 PMC8615182

[29] Kagami S Rizzo HL Lee JJ Koguchi Y Blauvelt A. Circulating Th17, Th22, and Th1 cells are increased in psoriasis. J Invest Dermatol. (2010) 130:1373–83. doi: 10.1038/jid.2009.399 PMC2892169 20032993

[30] Petrovic A Samuelsen VM Davies R Aarebrot AK Holmes T Sarkar I. Immune cell activity during anti-TNF treatment in patients with psoriasis and psoriatic arthritis. Clin Exp Immunol. (2024) 218(3):329–40. doi: 10.1093/cei/uxae070 PMC11557139 39121030

[31] Czarnowicki T Malajian D Shemer A Fuentes-Duculan J Gonzalez J Suárez-Fariñas M. Skin-homing and systemic T-cell subsets show higher activation in atopic dermatitis versus psoriasis. J Allergy Clin Immunol. (2015) 136:208–11. doi: 10.1016/j.jaci.2015.03.032 25936564

[32] Farrera C Melchiotti R Petrov N Teng KWW Wong MT Loh CY. T-cell phenotyping uncovers systemic features of atopic dermatitis and psoriasis. J Allergy Clin Immunol. (2020) 145:1021–1025.e15. doi: 10.1016/j.jaci.2019.11.030 31812571

[33] Yu Y Chen Z Wang Y Li Y Lu J Cui L. Infliximab modifies regulatory T cells and co-inhibitory receptor expression on circulating T cells in psoriasis. Int Immunopharmacol. (2021) 96:107722. doi: 10.1016/j.intimp.2021.107722 33965878

[34] Lanna C Mancini M Gaziano R Cannizzaro MV Galluzzo M Talamonti M. Skin immunity and its dysregulation in psoriasis. Cell Cycle. (2019) 18:2581–9. doi: 10.1080/15384101.2019.1653099 PMC6773242 31416396

[35] Vaishnav J Khan F Yadav M Parmar N Buch H Jadeja SD. V-set domain containing T-cell activation inhibitor-1 (VTCN1): A potential target for the treatment of autoimmune diseases. Immunobiology. (2022) 227:152274. doi: 10.1016/j.imbio.2022.152274 36095871

[36] Abrams JR Kelley SL Hayes E Kikuchi T Brown MJ Kang S. Blockade of T lymphocyte costimulation with cytotoxic T lymphocyte–associated antigen 4–immunoglobulin (CTLA4Ig) reverses the cellular pathology of psoriatic plaques, including the activation of keratinocytes, dendritic cells, and endothelial cells. J Exp Med. (2000) 192:681–94. doi: 10.1084/jem.192.5.681 PMC2193278 10974034

[37] Iannone F Lapadula G. The inhibitor of costimulation of T cells: abatacept. J Rheumatol Supplement. (2012) 89:100–2. doi: 10.3899/jrheum.120257 22751606

[38] Harris KM Smilek DE Byron M Lim N Barry WT McNamara J. Effect of costimulatory blockade with abatacept after ustekinumab withdrawal in patients with moderate to severe plaque psoriasis: the PAUSE randomized clinical trial. JAMA Dermatol. (2021) 157:1306–15. doi: 10.1001/jamadermatol.2021.3492 PMC8515260 34643650

[39] Yao Y Zeng L Huang X Zhang J Zhang G Wang L. Role of co−inhibitory molecules in the treatment of psoriasis. Exp Ther Med. (2024) 27:1–7. doi: 10.3892/etm.2024.12497 38590557 PMC11000047

[40] Liu S Xu J Wu J. The role of co-signaling molecules in psoriasis and their implications for targeted treatment. Front Pharmacol. (2021) 12:717042. doi: 10.3389/fphar.2021.717042 34354596 PMC8329336

[41] Kim JH Choi YJ Lee BH Song M-Y Ban CY Kim J. Programmed cell death ligand 1 alleviates psoriatic inflammation by suppressing IL-17A production from programmed cell death 1–high T cells. J Allergy Clin Immunol. (2016) 137:1466–1476.e3. doi: 10.1016/j.jaci.2015.11.021 26824999

[42] Sgambelluri F Diani M Altomare A Frigerio E Drago L Granucci F. A role for CCR5+ CD4 T cells in cutaneous psoriasis and for CD103+ CCR4+ CD8 Teff cells in the associated systemic inflammation. J Autoimmun. (2016) 70:80–90. doi: 10.1016/j.jaut.2016.03.019 27068801

[43] Bahri R Bulfone-Paus S. Mast cell activation test (MAT). Basophils Mast Cells: Methods Protoc. (2020) p:227–38. doi: 10.1007/978-1-0716-0696-4_19 32766980

[44] Sangineto M Graziano G D’Amore S Salvia R Palasciano G Sabba C. Identification of peculiar gene expression profile in peripheral blood mononuclear cells (PBMC) of celiac patients on gluten free diet. PloS One. (2018) 13:e0197915. doi: 10.1371/journal.pone.0197915 29795662 PMC5967809

[45] McCarthy DJ Chen Y Smyth GK. Differential expression analysis of multifactor RNA-Seq experiments with respect to biological variation. Nucleic Acids Res. (2012) 40:4288–97. doi: 10.1093/nar/gks042 PMC3378882 22287627

[46] Priyadarssini M Divya Priya D Indhumathi S Rajappa M Chandrashekar L Thappa D. Immunophenotyping of T cells in the peripheral circulation in psoriasis. Br J Biomed Sci. (2016) 73:174–9. doi: 10.1080/09674845.2016.1207869 27477596

[47] Koreck A Suranyi A Szöny B Farkas A Bata-Csörgö Z Kemeny L. CD3+ CD56+ NK T cells are significantly decreased in the peripheral blood of patients with psoriasis. Clin Exp Immunol. (2002) 127:176–82. doi: 10.1046/j.1365-2249.2002.01721.x PMC1906288 11882050

[48] Solberg SM Aarebrot AK Sarkar I Petrovic A Sandvik LF Bergum B. Mass cytometry analysis of blood immune cells from psoriasis patients on biological therapy. Eur J Immunol. (2021) 51:694–702. doi: 10.1002/eji.202048857 33226128

[49] Guo R Zhang T Meng X Lin Z Lin J Gong Y. Lymphocyte mass cytometry identifies a CD3–CD4+ cell subset with a potential role in psoriasis. JCI insight. (2019) 4:e125306. doi: 10.1172/jci.insight.125306 30747724 PMC6483065

[50] Diani M Galasso M Cozzi C Sgambelluri F Altomare A Cigni C. Blood to skin recirculation of CD4+ memory T cells associates with cutaneous and systemic manifestations of psoriatic disease. Clin Immunol. (2017) 180:84–94. doi: 10.1016/j.clim.2017.04.001 28392462

[51] Wang F Wang Y Wang L Wang T Bai Y. TIGIT expression levels on CD4+ T cells are correlated with disease severity in patients with psoriasis. Clin Exp Dermatol. (2018) 43:675–82. doi: 10.1111/ced.13414 29512851

[52] Jacobs ME Pouw JN Olde Nordkamp MA Radstake TR Leijten EF Boes M. DNAM1 and TIGIT balance the T cell response, with low T cell TIGIT expression corresponding to inflammation in psoriatic disease. Immunotherapy Adv. (2021) 1:ltaa004. doi: 10.1093/immadv/ltaa004 PMC9585685 36284900

[53] Owczarczyk-Saczonek A Krajewska-Włodarczyk M Kasprowicz-Furmańczyk M Placek W. Immunological memory of psoriatic lesions. Int J Mol Sci. (2020) 21:625. doi: 10.3390/ijms21020625 31963581 PMC7014148

[54] Olsen I Sollid LM. Pitfalls in determining the cytokine profile of human T cells. J immunological Methods. (2013) 390:106–12. doi: 10.1016/j.jim.2013.01.015 23416458

[55] Duraiswamy J Ibegbu CC Masopust D Miller JD Araki K Doho GH. Phenotype, function, and gene expression profiles of programmed death-1hi CD8 T cells in healthy human adults. J Immunol. (2011) 186:4200–12. doi: 10.4049/jimmunol.1001783 PMC3723805 21383243

[56] Sotsios Y Blair P Westwick J Ward SG. Disparate effects of phorbol esters, CD3 and the costimulatory receptors CD2 and CD28 on RANTES secretion by human T lymphocytes. Immunology. (2000) 101:30–7. doi: 10.1046/j.1365-2567.2000.00072.x PMC2327056 11012750

[57] Baumgaertner P Sankar M Herrera F Benedetti F Barras D Thierry A-C. Unsupervised analysis of flow cytometry data in a clinical setting captures cell diversity and allows population discovery. Front Immunol. (2021) 12:633910. doi: 10.3389/fimmu.2021.633910 33995353 PMC8119773

[58] Yeung H Takeshita J Mehta NN Kimmel SE Ogdie A Margolis DJ. Psoriasis severity and the prevalence of major medical comorbidity: a population-based study. JAMA Dermatol. (2013) 149:1173–9. doi: 10.1001/jamadermatol.2013.5015 PMC3800487 23925466

[59] Armstrong AW Mehta MD Schupp CW Gondo GC Bell SJ Griffiths CE. Psoriasis prevalence in adults in the United States. JAMA Dermatol. (2021) 157:940–6. doi: 10.1001/jamadermatol.2021.2007 PMC8246333 34190957

[60] Armstrong AW Schupp C Bebo B. Psoriasis comorbidities: results from the National Psoriasis Foundation surveys 2003 to 2011. Dermatology. (2012) 225:121–6. doi: 10.1159/000342180 23108113

[61] Zecevic-Pasic L Dzananovic N Gojak R Tihic-Kapidzic S Hasanefendic B Begovic E. Psoriasis area and severity index (PASI) objectivisation by flow cytometry analysis of major lymphocytes subsets. Acta Informatica Med. (2023) 31:206. doi: 10.5455/aim.2023.31.206-210 PMC10540746 37781498

[62] Langewouters A Van Erp P De Jong E Van De Kerkhof P. Lymphocyte subsets in peripheral blood of patients with moderate-to-severe versus mild plaque psoriasis. Arch Dermatol Res. (2008) 300:107–13. doi: 10.1007/s00403-007-0819-9 PMC2254658 18157542

[63] Dunphy S Sweeney C Kelly G Tobin A Kirby B Gardiner C. Natural killer cells from psoriasis vulgaris patients have reduced levels of cytotoxicity associated degranulation and cytokine production. Clin Immunol. (2017) 177:43–9. doi: 10.1016/j.clim.2015.10.004 26477484

[64] Villanova F Flutter B Tosi I Grys K Sreeneebus H Perera GK. Characterization of innate lymphoid cells in human skin and blood demonstrates increase of NKp44+ ILC3 in psoriasis. J Invest Dermatol. (2014) 134:984–91. doi: 10.1038/jid.2013.477 PMC3961476 24352038

[65] Louati N Rekik T Menif H Gargouri J. Blood lymphocyte T subsets reference values in blood donors by flow cytometry. etablissement des valeurs de référence des lymphocytes t chez les donneurs de sang parcytométrie en flux. La Tunisie Medicale. (2019) 97:327–34. 31539091

[66] Lu J Ahmad R Nguyen T Cifello J Hemani H Li J. Heterogeneity and transcriptome changes of human CD8+ T cells across nine decades of life. Nat Commun. (2022) 13:5128. doi: 10.1038/s41467-022-32869-x 36050300 PMC9436929

[67] Machura E Mazur B Kwiecień J Karczewska K. Intracellular production of IL-2, IL-4, IFN-γ, and TNF-α by peripheral blood CD3+ and CD4+ T cells in children with atopic dermatitis. Eur J Pediatr. (2007) 166:789–95. doi: 10.1007/s00431-006-0319-5 17120040

[68] Seidel J Vukmanovic-Stejic M Muller-Durovic B Patel N Fuentes-Duculan J Henson S. Skin resident memory CD8+ T cells are phenotypically and functionally distinct from circulating populations and lack immediate cytotoxic function. Clin Exp Immunol. (2018) 194:79–92. doi: 10.1111/cei.13189 30030847 PMC6156810

[69] Appay V van Lier RA Sallusto F Roederer M. Phenotype and function of human T lymphocyte subsets: consensus and issues. Cytometry Part A. (2008) 73:975–83. doi: 10.1002/cyto.a.v73a:11 18785267

[70] Sahir F Mateo JM Steinhoff M Siveen KS. Development of a 43 color panel for the characterization of conventional and unconventional T-cell subsets, B cells, NK cells, monocytes, dendritic cells, and innate lymphoid cells using spectral flow cytometry. Cytometry Part A. (2024) 105:404–10. doi: 10.1002/cyto.a.v105.5 PMC11497249 33336868

[71] Kizmaz MA Simsek A Bozkurt T Cagan E Dombaz F Tezcan G. Effector memory T cell subset CD45RA– CCR7– CD27– CD28– EM3 increases in direct proportion to the disease severity of COVID-19. Scandinavian J Immunol. (2023) 97:e13217. doi: 10.1111/sji.13217

[72] Finlay AY. Current severe psoriasis and the rule of tens. Br J Dermatol. (2005) 152:861–7. doi: 10.1111/j.1365-2133.2005.06502.x 15888138

[73] Andres-Ejarque R Ale HB Grys K Tosi I Solanky S Ainali C. Enhanced NF-κB signaling in type-2 dendritic cells at baseline predicts non-response to adalimumab in psoriasis. Nat Commun. (2021) 12:4741. doi: 10.1038/s41467-021-25066-9 34362923 PMC8346545

[74] Kim J Bissonnette R Lee J da Rosa JC Suárez-Fariñas M Lowes MA. The spectrum of mild to severe psoriasis vulgaris is defined by a common activation of IL-17 pathway genes, but with key differences in immune regulatory genes. J Invest Dermatol. (2016) 136:2173–82. doi: 10.1016/j.jid.2016.04.032 27185339

[75] Vissers W Arndtz C Muys L Van Erp P De Jong E Van De Kerkhof P. Memory effector (CD45RO+) and cytotoxic (CD8+) T cells appear early in the margin zone of spreading psoriatic lesions in contrast to cells expressing natural killer receptors, which appear late. Br J Dermatol. (2004) 150:852–9. doi: 10.1111/j.1365-2133.2004.05863.x 15149496

[76] Casciano F Diani M Altomare A Granucci F Secchiero P Banfi G. CCR4+ skin-tropic phenotype as a feature of central memory CD8+ T cells in healthy subjects and psoriasis patients. Front Immunol. (2020) 11:529. doi: 10.3389/fimmu.2020.00529 32318062 PMC7147166

[77] Reali E Ferrari D. From the skin to distant sites: T cells in psoriatic disease. Int J Mol Sci. (2023) 24:15707. doi: 10.3390/ijms242115707 37958689 PMC10648543

[78] Davison S Allen M Harmer A Vaughan R Barker J. Increased T-cell receptor Vβ2 chain expression in skin homing lymphocytes in psoriasis. Br J Dermatol. (1999) 140:845–8. doi: 10.1046/j.1365-2133.1999.02813.x 10354020

[79] Teraki Y Hotta T Shiohara T. Increased circulating skin-homing cutaneous lymphocyte-associated antigen (CLA)+ type 2 cytokine-producing cells, and decreased CLA+ type 1 cytokine-producing cells in atopic dermatitis. Br J Dermatol. (2000) 143:373–8. doi: 10.1046/j.1365-2133.2000.03665.x 10951148

[80] Sigmundsdóttir H Gudjonsson J Jónsdóttir I Ludviksson B Valdimarsson H. The frequency of CLA+ CD8+ T cells in the blood of psoriasis patients correlates closely with the severity of their disease. Clin Exp Immunol. (2001) 126:365–9. doi: 10.1046/j.1365-2249.2001.01688.x PMC1906191 11703383

[81] Sigmundsdottir H Johnston A Gudjonsson JE Bjarnason B Valdimarsson H. Methotrexate markedly reduces the expression of vascular E-selectin, cutaneous lymphocyte-associated antigen and the numbers of mononuclear leucocytes in psoriatic skin. Exp Dermatol. (2004) 13:426–34. doi: 10.1111/j.0906-6705.2004.00177.x 15217363

[82] Zhang Y Lin Y Wang L Sun X Dang E Xue K. CD8αα+ T cells exert a pro-inflammatory role in patients with psoriasis. Skin Health Dis. (2021) 1:e64. doi: 10.1002/ski2.64 35663772 PMC9060015

[83] Bosè F Capsoni F Molteni S Raeli L Diani M Altomare A. Differential expression of interleukin-2 by anti-CD3-stimulated peripheral blood mononuclear cells in patients with psoriatic arthritis and patients with cutaneous psoriasis. Clin Exp Dermatol. (2014) 39:385–90. doi: 10.1111/ced.12251 24772485

[84] Hou H Zhou Y Yu J Mao L Bosco MJ Wang J. Establishment of the reference intervals of lymphocyte function in healthy adults based on IFN-γ secretion assay upon phorbol-12-myristate-13-acetate/ionomycin stimulation. Front Immunol. (2018) 9:172. doi: 10.3389/fimmu.2018.00172 29467761 PMC5808316

[85] Manescu IB Manu DR Serban GM Dobreanu M. Variability of stimulated T-cells secretory profile in healthy subjects. Rev Romana Medicina Laborator. (2020) 28:75–89. doi: 10.2478/rrlm-2020-0004

[86] Xu X Davelaar N Mus AM Asmawidjaja PS Hazes JM Baeten DL. Interleukin-17A is produced by CD4+ but not CD8+ T cells in synovial fluid following T cell receptor activation and regulates different inflammatory mediators compared to tumor necrosis factor in a model of psoriatic arthritis synovitis. Arthritis Rheumatol. (2020) 72:1303–13. doi: 10.1002/art.41271 PMC7497075 32243724

[87] Sobhan M Ghasemi Basir H Seif Rabiee M Shadfar F. Serum level of interleukin-17 in patients with psoriasis. Immunopathol Persa. (2022), e29269. doi: 10.34172/ipp.2022.29269

[88] Lovato P Norsgaard H Tokura Y Røpke MA. Calcipotriol and betamethasone dipropionate exert additive inhibitory effects on the cytokine expression of inflammatory dendritic cell–Th17 cell axis in psoriasis. J Dermatol Sci. (2016) 81:153–64. doi: 10.1016/j.jdermsci.2015.12.009 26794805

[89] Jain H Devabattula G Bhat A Dalvi H Rangaraj N Godugu C. Topical delivery of Bruton’s tyrosine kinase inhibitor and curcumin-loaded nanostructured lipid carrier gel: Repurposing strategy for the psoriasis management. Pharmaceutical Dev Technol. (2022) 27:975–88. doi: 10.1080/10837450.2022.2142610 36330998

[90] Alsabbagh MM. Cytokines in psoriasis: From pathogenesis to targeted therapy. Hum Immunol. (2024) 85:110814. doi: 10.1016/j.humimm.2024.110814 38768527

[91] Hwang YJ Jung HJ Kim MJ Roh NK Jung JW Lee YW. Serum levels of LL-37 and inflammatory cytokines in plaque and guttate psoriasis. Mediators Inflammation. (2014) 2014:268257. doi: 10.1155/2014/268257 PMC4150496 25197165

[92] Jacob SE Nassiri M Kerdel FA Vincek V. Simultaneous measurement of multiple Th1 and Th2 serum cytokines in psoriasis and correlation with disease severity. Mediators Inflammation. (2003) 12:309–13. doi: 10.1080/09629350310001619753 PMC1781622 14760939

[93] Tsoi LC Patrick MT Shuai S Sarkar MK Chi S Ruffino B. Cytokine responses in nonlesional psoriatic skin as clinical predictor to anti-TNF agents. J Allergy Clin Immunol. (2022) 149:640–649.e5. doi: 10.1016/j.jaci.2021.07.024 34343561 PMC9451046

[94] Arican O Aral M Sasmaz S Ciragil P. Serum levels of TNF-α, IFN-γ, IL-6, IL-8, IL-12, IL-17, and IL-18 in patients with active psoriasis and correlation with disease severity. Mediators Inflammation. (2005) 2005:273–9. doi: 10.1155/MI.2005.273 PMC1533889 16258194

[95] Ortega C Fernández-A S Carrillo JM Romero P Molina IJ Moreno JC. IL-17-producing CD8+ T lymphocytes from psoriasis skin plaques are cytotoxic effector cells that secrete Th17-related cytokines. J leukocyte Biol. (2009) 86:435–43. doi: 10.1189/JLB.0109046 19487306

[96] Prince HE York J Jensen ER. Phenotypic comparison of the three populations of human lymphocytes defined by CD45RO and CD45RA expression. Cell Immunol. (1992) 145:254–62. doi: 10.1016/0008-8749(92)90329-N 1451178

[97] Eder L Caucheteux SM Afiuni-Zadeh S Croitoru D Krizova A Limacher JJ. Imaging mass cytometry in psoriatic disease reveals immune profile heterogeneity in skin and synovial tissue. J Invest Dermatol. (2024) 10:S0022-202X(24)02180-8. doi: 10.1016/j.jid.2024.08.039 39393504

[98] Voillet V Buggert M Slichter CK Berkson JD Mair F Addison MM. Human MAIT cells exit peripheral tissues and recirculate via lymph in steady state conditions. JCI insight. (2018) 3:e98487. doi: 10.1172/jci.insight.98487 29618662 PMC5928862

[99] Sun X Lin X Li Z Wu H. A comprehensive comparison of supervised and unsupervised methods for cell type identification in single-cell RNA-seq. Briefings Bioinf. (2022) 23:bbab567. doi: 10.1093/bib/bbab567 PMC8921620 35021202

[100] Díaz-Fernández S Villar-Hernández R Stojanovic Z Fernández M Galvão MLDS Tolosa G. Study of CD27, CD38, HLA-DR and Ki-67 immune profiles for the characterization of active tuberculosis, latent infection and end of treatment. Front Microbiol. (2022) 13:885312. doi: 10.3389/fmicb.2022.885312 35935194 PMC9354672

[101] Günter M Mueller KAL Salazar MJ Gekeler S Prang C Harm T. Immune signature of patients with cardiovascular disease predicts increased risk for a severe course of COVID-19. Eur J Immunol. (2023) p:2451145. doi: 10.1002/eji.202451145 39094122

[102] Fox A Dutt TS Karger B Rojas M Obregón-Henao A Anderson GB. Cyto-feature engineering: A pipeline for flow cytometry analysis to uncover immune populations and associations with disease. Sci Rep. (2020) 10:7651. doi: 10.1038/s41598-020-64516-0 32377001 PMC7203241

[103] Melsen JE van Ostaijen-Ten Dam MM Lankester AC Schilham MW van den Akker EB. A comprehensive workflow for applying single-cell clustering and pseudotime analysis to flow cytometry data. J Immunol. (2020) 205:864–71. doi: 10.4049/jimmunol.1901530 32591399

[104] Palit S Heuser C De Almeida GP Theis FJ Zielinski CE. Meeting the challenges of high-dimensional single-cell data analysis in immunology. Front Immunol. (2019) 10:1515. doi: 10.3389/fimmu.2019.01515 31354705 PMC6634245

[105] Pouyan MB Jindal V Birjandtalab J Nourani M. Single and multi-subject clustering of flow cytometry data for cell-type identification and anomaly detection. BMC Med Genomics. (2016) 9:99–110. doi: 10.1186/s12920-016-0201-x PMC4980779 27510222

[106] Ho PY Barton A Worthington J Thomson W Silman AJ Bruce IN. HLA-Cw6 and HLA-DRB1* 07 together are associated with less severe joint disease in psoriatic arthritis. Ann rheumatic Dis. (2007) 66:807–11. doi: 10.1136/ard.2006.064972 PMC1954651 17223660

[107] Griffiths CE Barnes MR Burden AD Nestle FO Reynolds NJ Smith CH. Establishing an academic-industrial stratified medicine consortium: psoriasis stratification to optimize relevant therapy. J Invest Dermatol. (2015) 135 (12):2903–7. doi: 10.1038/jid.2015.286 26569580

[108] Reid C Cordingley L Warren RB Griffiths CE. Progress to date in advancing stratified medicine in psoriasis. Am J Clin Dermatol. (2020) 21:619–26. doi: 10.1007/s40257-020-00533-z 32607944

