# Supplementary material for: Circulating CD8 T cells from patients with mild-to-moderate psoriasis are functionally impaired
Source: Front Immunol. 2025 May 5;16:1585378. doi: 10.3389/fimmu.2025.1585378 (PMC12086172; doi:10.3389/fimmu.2025.1585378)
Supplement: Supplementary file 1 [file DataSheet1.docx]

**Supplemental information Chen et al.**

1. **Supplemental methods**
   1. **Patients**

This study was approved by the London Bridge Research Ethics Committee (REC number: 06/Q0704/18). Peripheral blood samples were collected from 7 psoriasis patients (mean age 47.28 ± 19.97; range 33-65) at Guy’s and St Thomas’ Hospital, London, UK, after obtaining informed consent. All patients had received topical therapies before sampling, with a mean Psoriasis Area and Severity Index (PASI) score of 6.7 ± 1.6. According to the PASI score, two subjects were considered to have mild psoriasis (PASI < 5) and five moderate psoriasis (PASI 5-10)(1). As a control cohort, 11 healthy donors without underlying disease were recruited from the NHS Blood and Transplant Unit (Manchester, UK) after they provided informed consent. Samples were obtained under a material transfer agreement and used following a protocol approved by the University of Manchester research ethics committee (UREC ref: 2018-2696-5711).

- 1. **Flow cytometry manual gating strategy**

Analysed peripheral blood mononuclear cells (PBMCs) were gated on FSC-A/SSC-A parameters. After removing cellular debris, non-viable cells, and doublets, T cells (CD3+CD56-), NK cells (CD3-CD56-), and NKT cells (CD3+CD56+) were identified using their respective lineage markers. T cells were further categorized into CD8- and CD8+ subsets. Within the CD8+ T cell population, TCRVα7.2+CD161+ MAIT cells were identified, and the remaining MAIT-CD8+ T cells were classified based on CD45RO and CD45RA expression. CD27 expression was used to differentiate naïve and effector CD8+ T cells within the naïve/effector subset and effector memory and central memory CD8+ T cells within the memory subset. The study also characterized CCR7+ central memory and CCR7- effector memory T cells from the memory CD8+ T cell population and CCR7+ naïve and CCR7- effector T cells from the double-negative CD8+ T cell subset. Cell populations were quantified as percentages of viable lymphocytes or CD8+ T cells, except for chemokine and co-signalling marker-expressing cells, which were reported as percentages of their respective parent populations. Fluorescence-minus-one (FMO) controls were utilized to establish positive gating for each parameter. A representative gating strategy is depicted in **Supplemental Figure 1B**.

- 1. **Unsupervised analysis workflow**

For phenotypic characterisation, all live single lymphocytes from all samples were exported from FlowJo Software, while for intracellular cytokine production, only T cells (CD3+CD56- cells) were exported. The concatenated FCS files for each sample replicate were uploaded to OMIQ, a cloud-based flow cytometry analytical platform (Dotmatics). First, scaling co-factors were adjusted manually for all fluorescent channels in each sample, fluorescence was converted to inverse hyperbolic sine values (asinh), and events were subsampled to the sample with the least number of events per panel. Flow cytometry samples were then batch-corrected using the CyCombine function(2). Subsequently, dimensionality reduction was performed using Uniform Manifold Approximation and Projection (UMAP) to visualise the different sub-populations of the cells. Following UMAP, unbiased clustering was performed using FlowSOM, which created meta-clusters grouping cell populations by shared recognised cell surface markers using the elbow method. A heatmap was generated with the median expression of the analysed cell markers to assign clusters to distinct cell populations. To adjust expression based on fluorescence controls, FMO controls from all subjects were pooled, and the lowest threshold for positivity was manually defined per each studied marker using FlowJo (**Supplemental Figures 7 and 8**). Fluorescence positivity thresholds were then converted to arcsinh values after dividing for the scaling co-factor and applied to the median expression for each identified cluster. If below the defined threshold, the values were set to 0. If above the specified threshold, the average expression was normalised between 1 (low expression) and 5 (high expression). Multiple rounds of clustering were done to exclude poorly performing markers, namely markers producing over-clustering, clustering on outliers and/or expression constantly below the positivity threshold in every identified cluster. If meta-clusters displayed identical markers expression, they were merged using Boolean OR gating. Raw counts and meta-cluster frequencies relative to the total number of analysed cells (percent of total) were then exported from OMIQ and used for downstream analyses.

1. **Supplemental results**
   1. **CCR7-based gating reveals significant differences in CD8 T effector memory frequencies in healthy compared to psoriasis patients**

For identifying naïve T cell subpopulations, gating strategies based on CD27 or CCR7 expression are often used, which display a high degree of correlation(3). Therefore, complementary to CD27, we conducted a CCR7-based gating analysis of CD8 T cell subsets to assess potential differences in subpopulation frequencies between strategies. CCR7-based gating revealed significant differences in the identification of CM and EM cells compared to CD27-based gating (**Supplemental Figure 1C**). However, consistent with CD27-based gating, the CCR7-based analysis showed no significant differences in the frequencies of CM, naïve and effector CD8 T cells. Interestingly, CD8 EM (CCR7-) T cells showed significantly lower frequency in psoriasis patients compared to healthy controls (*p*<0.05) (**Supplemental Figure 6**).

- 1. **Psoriasis does not produce significant changes in the expression of homing/trafficking markers by CD8 T cells using unsupervised clustering**

Unsupervised data analysis and clustering with FlowSOM identified 20 distinct cell clusters, of which two (Cluster 17 and 18) could not be assigned or associated with the cell populations identified in the supervised analysis. The generated meta-clusters included CD8 T cells (Cluster 02, 03, 04, 05, 07, 08, 11, 15), CD8- T cells (Cluster 01, 06, 09, 10, 13, 14, 19, 20) and NK cells (Cluster 16 and 21) (**Supplemental Figure 9A-C**). Among studied homing/trafficking markers, the median cluster expression of CD69, CLA and CXCR3 was below the positivity threshold set by FMO controls in each identified cluster (**Supplemental Figure 9C**).

We then assessed differences in the count abundance of identified clusters in PBMCs from psoriasis patients and healthy controls using EdgeR. We could detect significant differences between psoriasis patients and healthy controls in one cluster, representing a CD8- T cell population characterised by the co-expression of CD27 and CCR7 (Cluster 19, **Supplemental Figure 9D-E**). However, when analysing the frequency of this cluster relative to the total number of cells analysed, we could observe significant changes only in unadjusted analyses (unadjusted p value=0.023, FDR-adjusted p=0.43). Furthermore, when performing manual gating confirmation of the identified cluster, we could not confirm the significant difference between healthy and psoriasis subjects (**Supplemental Figure 9F**).

- 1. **Unsupervised clustering reveals differences in NKT cell abundance between healthy and psoriasis**

In the unsupervised analysis, upon excluding markers which were constantly below the threshold for positivity and/or caused over-clustering (i.e., CD134, PD-1, LIGHT, **Supplemental Figure 8**), 18 different cell clusters were identified. Among these, 4 were positive for BTLA and 11 clusters for CD28 (**Supplemental Figure 10A-C**). We analysed the differential count abundance of these clusters between psoriasis patients and healthy controls using EdgeR. The analysis showed significant differences in the abundance of two meta-clusters identifying CD8- and CD8 NKT cells (Cluster 03, CD3+CD8-CD56+CD45RA-CD45RO+CD27+CD28+; Cluster 13, CD3+CD8+CD56+CD45RA+CD45RO+CD27+CD28+) between psoriasis patients and healthy controls (**Supplemental Figure 10D-E**). However, when we analysed the cluster percentages relative to the total lymphocyte population, we observed statistically significant differences only in unadjusted analyses (Cluster 03 unadjusted p=0.005, FDR-adjusted p=0.087; Cluster 13 unadjusted p=0.016, FDR-adjusted p=0.143, **Supplemental Figure 10F**). Conversely, manual validation of found NKT clusters did not confirm the significant differences found in healthy and psoriasis patients (**Supplemental Figure 10G**).

- 1. **Unsupervised analyses highlight underrepresented CD8- and increased naïve CD8+ T cell subpopulations following stimulation**

In unsupervised analyses, cluster frequency analysis following anti-CD3/CD28 stimulation revealed six differentially expressed CD8- and one CD8+ T cell cluster in psoriasis patients compared to healthy. In particular, we observed a significant reduction of CD8-CD45RA-CD45RO-CD27+ T cells expressing IFNγ and low TNF (Cluster 07), CD8-CD45RA-CD45RO-CD27+CD161+ T cells expressing CD69 and Granzyme B (Cluster 13), and CD8-CD45RA-CD45RO-CD27+CD161+ T cells expressing CD69, Granzyme B (low expression), IL-17A, IL-17F and TNF (Cluster 14) in psoriasis compared to healthy subjects. Conversely, we observed increased frequency of a single CD8+ naïve T cell cluster co-expressing CD45RA and CD27 and negative for CD45RO in psoriasis patients (Cluster 15). Furthermore, the analysis of unstimulated and PMA/ION stimulated cells revealed significant changes in several CD8- T cell subpopulations, summarised in **Supplemental Table 5**.

Although showing similar trends, manual analysis did not corroborate the significant differences in the frequency of the identified clusters between psoriasis patients and healthy individuals (**Figure 4F-G**, additional data not shown).

- 1. **Unsupervised TNF-expressing CD8- T clusters negatively correlate with PASI scores**

We performed correlation and linear regression analyses between the frequency of found clusters using unsupervised clustering and PASI scores of psoriasis patients. We could not observe significant correlations in any CD8 T clusters identified ex vivo or following stimulation with PMA/ION or anti-CD3/CD28. However, we could observe significant correlations with several CD8- T cell clusters (**Supplemental Table 6**). PASI scores negatively correlated with circulating CD45RA-CD45RO+CD103+CCR4+ CD8- T cells (Cluster 13) and with two TNF+ clusters, namely CD45RA-CD45RO-CD27+CD161+TNF+ (Cluster 6) and CD45RA-CD45RO-CD27+IFNγ+TNF+ CD8- T cells (Cluster 2) in unstimulated and anti-CD3/CD28-stimulated conditions, respectively.

**References**

1. Martins C, Darrigade AS, Jacquemin C, Barnetche T, Taieb A, Ezzedine K, et al. Phenotype and function of circulating memory T cells in human vitiligo. British Journal of Dermatology. 2020;183(5):899-908.

2. Pedersen CB, Dam SH, Barnkob MB, Leipold MD, Purroy N, Rassenti LZ, et al. cyCombine allows for robust integration of single-cell cytometry datasets within and across technologies. Nature communications. 2022;13(1):1698.

3. Ferrando-Martinez S, Ruiz-Mateos E, Leal M. CD27 and CCR7 expression on naive T cells, are both necessary? Immunology letters. 2009;127(2):157-8.
