# Supplementary material for: Circulating CD8 T cells from patients with mild-to-moderate psoriasis are functionally impaired
Source: Front Immunol. 2025 May 5;16:1585378. doi: 10.3389/fimmu.2025.1585378 (PMC12086172; doi:10.3389/fimmu.2025.1585378)
Supplement: Supplementary file 2 [file DataSheet2.docx]

# Supplemental Figures

**
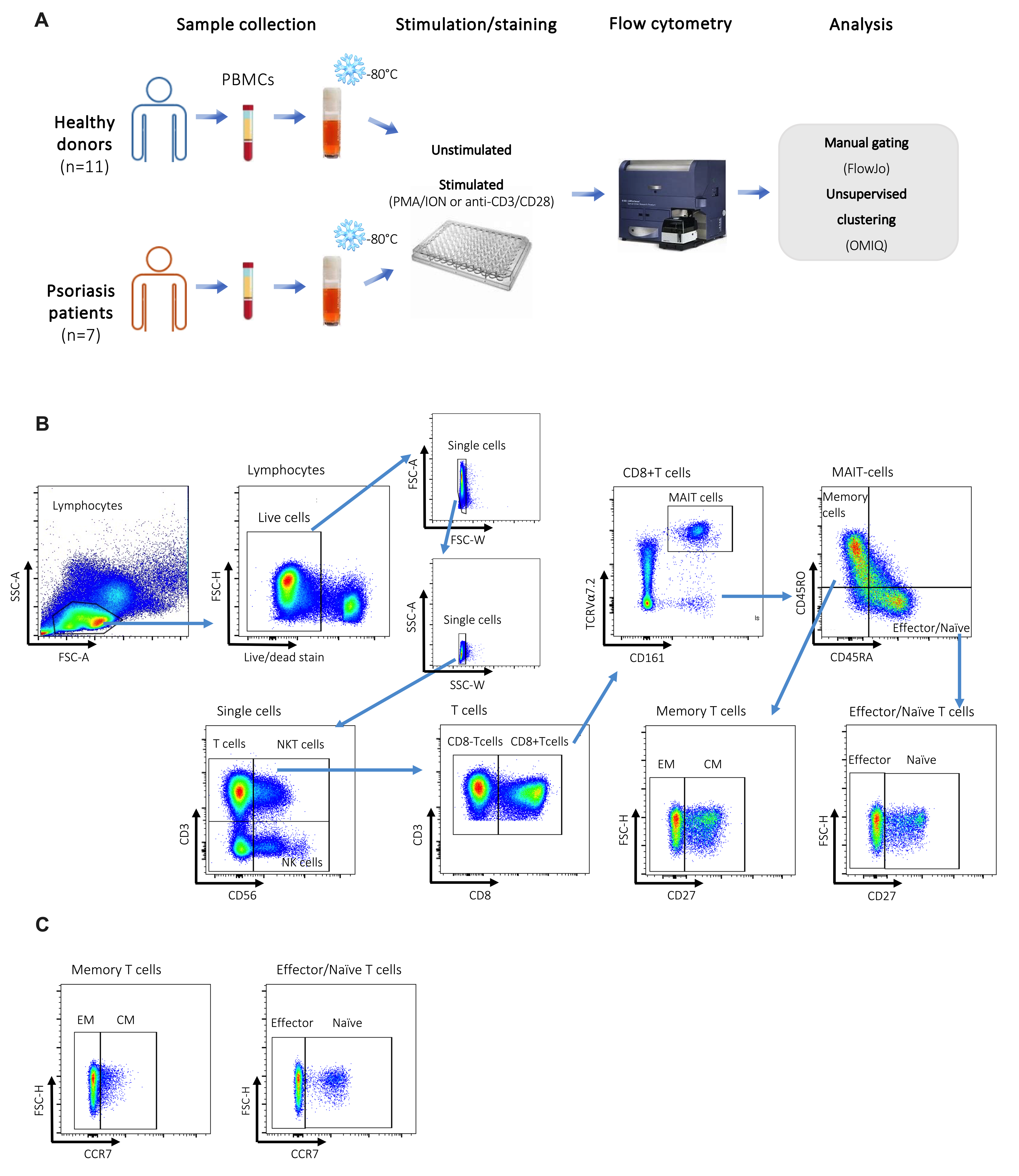
Supplemental Figure 1. Experimental flow of the study and manual gating strategy.** Peripheral blood mononuclear cells (PBMCs) were isolated from individuals with mild-to-moderate psoriasis (n=7) and healthy controls (n=11). (**A**) The experimental design and (**B**) the flow cytometry gating strategy used for the analysis of immune cell populations in a representative healthy donor sample are shown. Abbreviations: CM, central memory T cells; Effector, effector T cells; EM, effector memory T cells; MAIT, mucosal-associated invariant T; Naïve, naïve T cells; NK, natural killer.


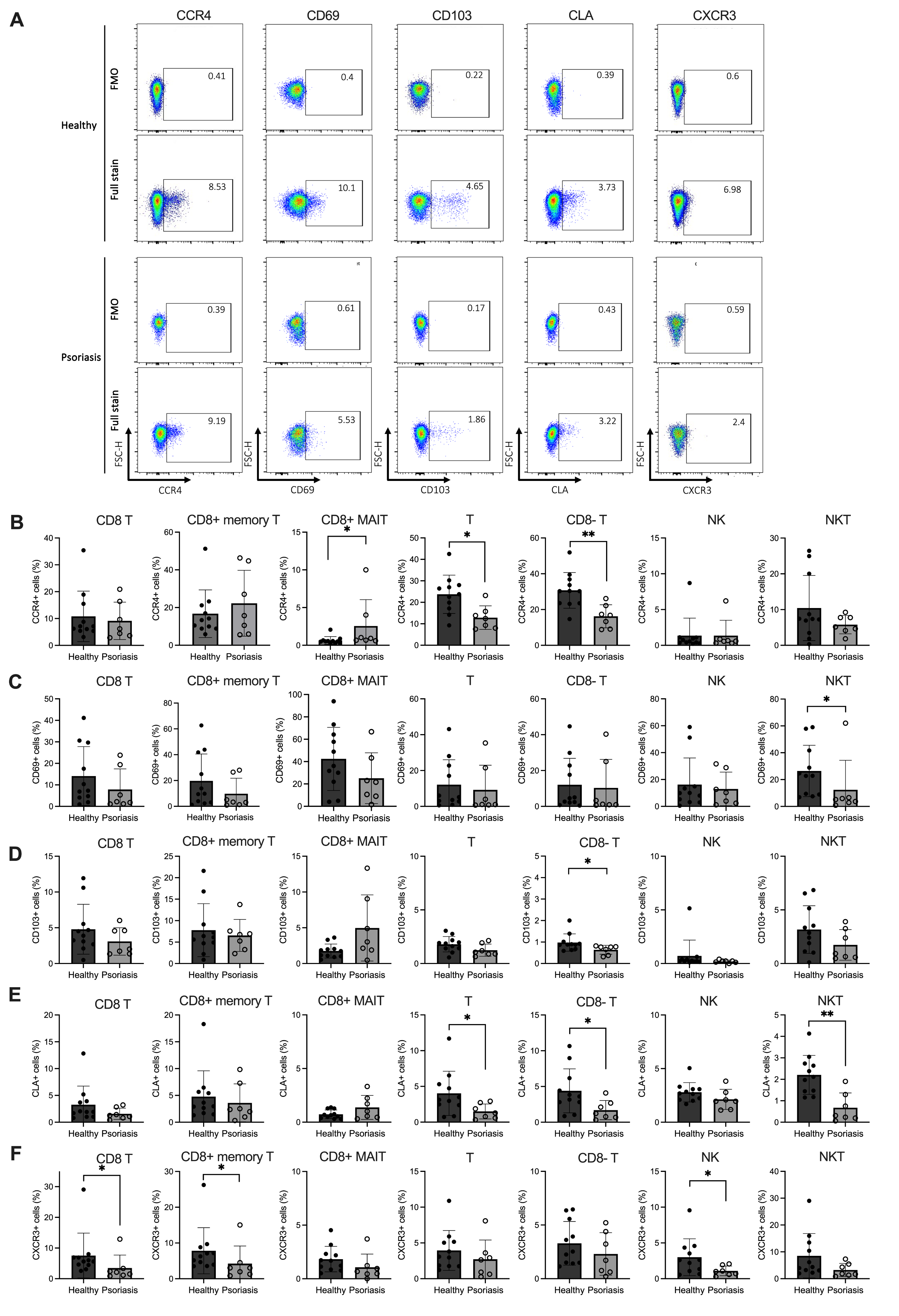


**Supplemental Figure 2. Expression of skin homing markers by CD8 T cells and other immune cell subsets in psoriasis and healthy subjects.** Peripheral blood mononuclear cells (PBMCs) were isolated from individuals with mild-to-moderate psoriasis (n=7) and healthy controls (n=11) and analysed in flow cytometry. (**A**) Representative flow plot of skin homing marker expression on CD8 T cells. Bar plots show the relative percentages (y-axis) of (**B**) CCR4, (**C**) CD69, (**D**) CD103, (**E**) CLA and (**F**) CXCR3-expressing cells within CD8 T cells and other immune cell subsets. Significant differences were analysed using the Mann-Whitney U test. **p* value < 0.05, **p < 0.01. Bar plots represent mean value ± SD.

Abbreviations: NK, natural killer cells; NKT, natural killer T cells; MAIT, mucosal-associated invariant T cells.


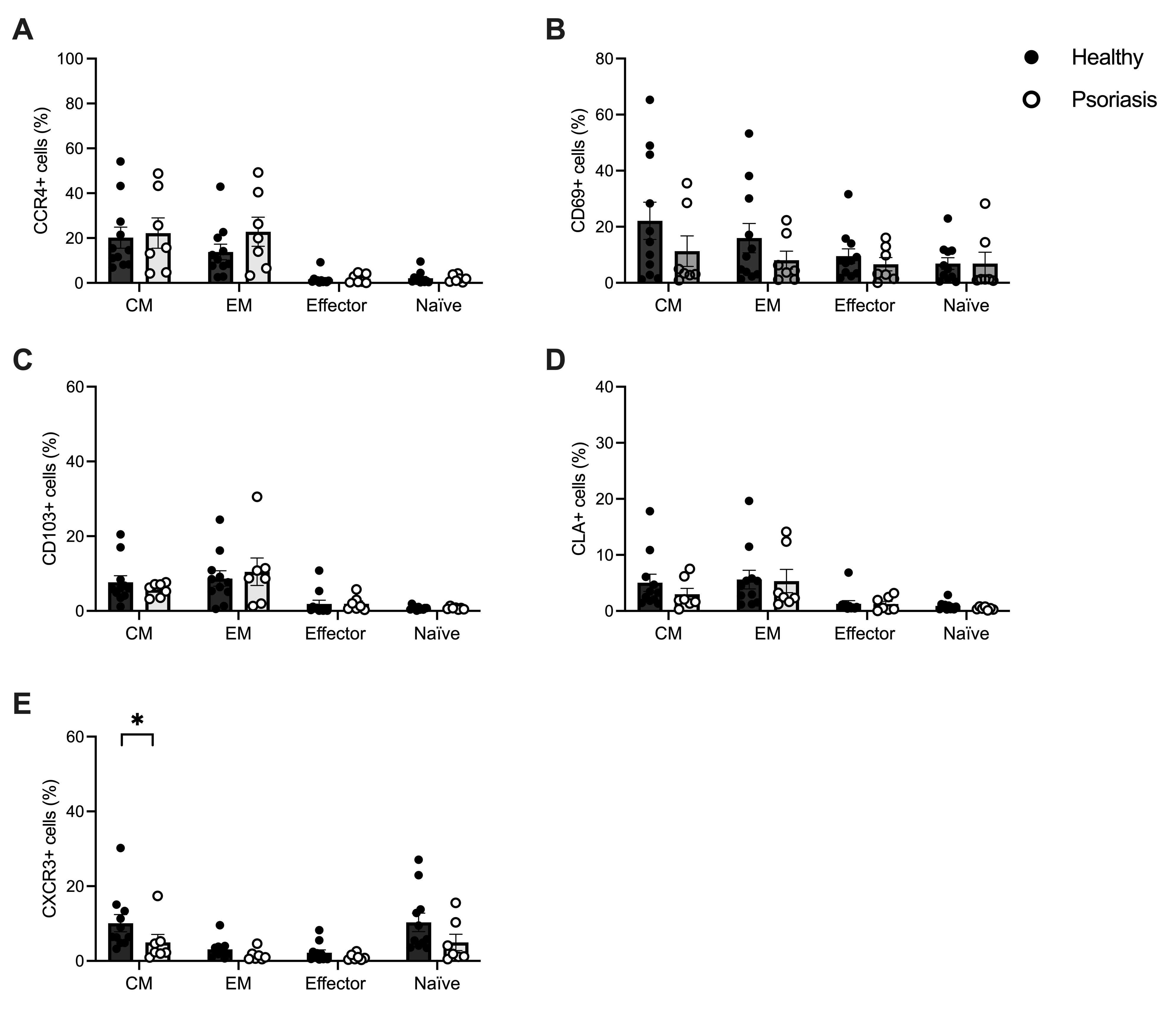


**Supplemental Figure 3. Expression of skin homing markers by CD8 T cell subsets.** Peripheral blood mononuclear cells (PBMCs) were obtained from individuals with mild-to-moderate psoriasis (n=7) and healthy controls (n=11) and analysed in flow cytometry. Bar plots show the relative percentages (y-axis) of (**A**) CCR4, (**B**) CD69, (**C**) CD103, (**D**) CLA and (**E**) CXCR3-expressing cells within CD8 CM, EM, Effector and Naïve T cells subsets. Significant differences were analysed using the Mann-Whitney U test. **p* value < 0.05. Bar plots represent mean value ± SD.

Abbreviations: CM, central memory T cells; EM, effector memory T cells; Effector, effector T cells; Naïve, naïve T cells.


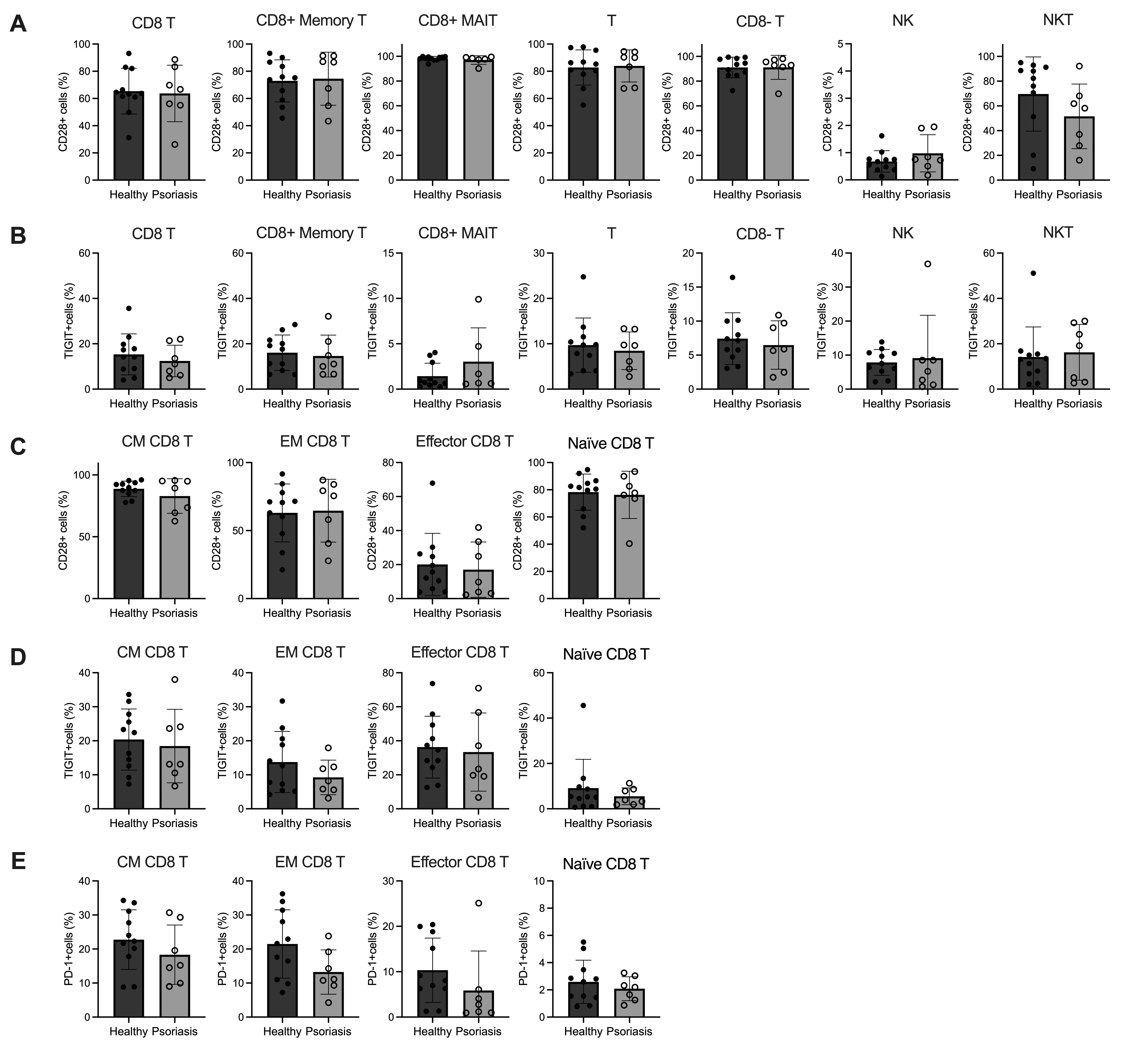


**Supplemental Figure 4. Expression of CD28 and TIGIT by circulating immune cell subsets is unchanged in patients with mild-to-moderate psoriasis.** Peripheral blood mononuclear cells (PBMCs) from psoriasis (n=7) and healthy individuals (n=11) were analysed in flow cytometry. Bar plots show the relative percentages (y-axis) of (**A**) CD28 and (**B**) TIGIT-expressing CD8 T cells and other immune cell subsets, (**C**) CD28, (**D**) TIGIT and (**E**) PD-1-expressing circulating CD8 CM, EM, Eff and Naïve T cells in mild-to-moderate psoriasis compared to healthy individuals. Differences were calculated using the Mann-Whitney U test. Bar plots represent mean value ± SD.

Abbreviations: CM, central memory T cells; EM, effector memory T cells; Effector, effector T cells; Naïve, naïve T cells; MAIT, mucosal-associated invariant T cells; NK, natural killer cells; NKT, natural killer T cells.


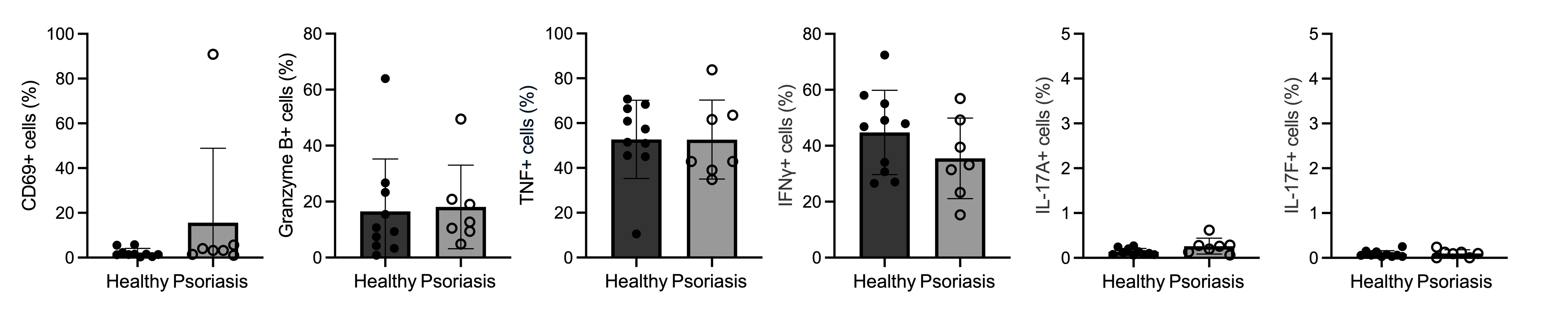


**Supplemental Figure 5. The expression of pro-inflammatory mediators by CD8 T cells in mild-to-moderate psoriasis is similar to healthy subjects.** Peripheral blood mononuclear cells (PBMCs) were isolated from psoriasis patients (n=7) and healthy controls (n=10). PBMCs were stimulated with phorbol 12-myristate 13-acetate plus ionomycin, and the membrane and intracellular expression of mediators were analysed using flow cytometry. Bar plots show the percentage of CD8+ T cells expressing CD69, Granzyme B, TNF, IFNγ, IL-17A or IL-17F. Differences between groups were calculated using the Mann-Whitney U test. Bar plots represent mean value ± SD.

**
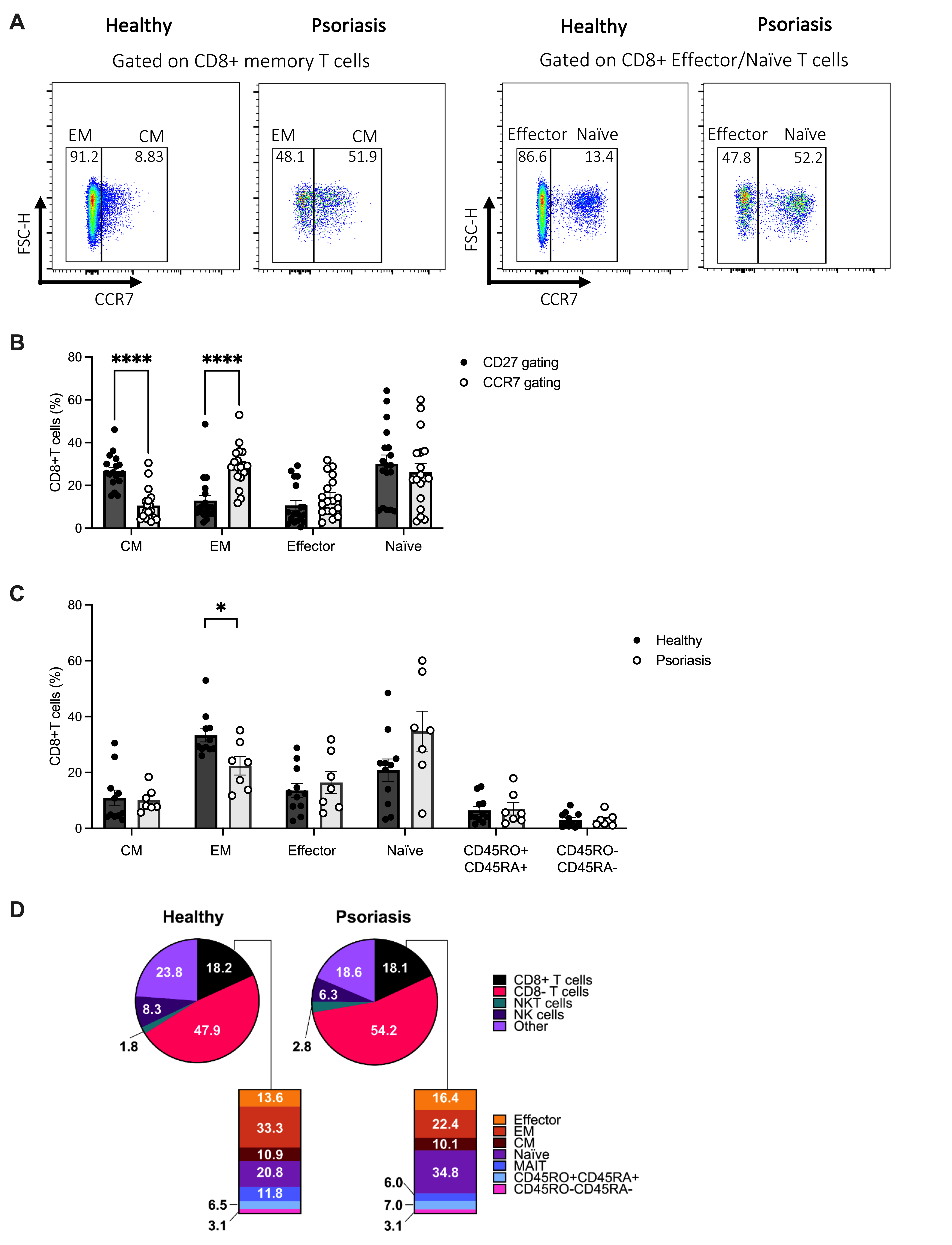
**

**Supplemental Figure 6. Differences in the immunophenotype of patients with mild-to-moderate psoriasis and healthy subjects using CD27- and CCR7-based gating strategies.** Cell surface marker combinations for defining cell subtypes were summarized in Supplementary Table 3. (**A**) Representative flow plots of CD8 T cell gating strategy from one healthy control (left panel) and one psoriasis patient (right panel). MAIT CD8 cells (TCRVα7.2+CD161+) were gated out from other CD8 T subpopulations, further characterised using the following combinations: Effector (CD45RA+CD45RO-CCR7-, Eff), Effector Memory (CD45RA-CD45RO+CCR7-, EM), central memory (CD45RA-CD45RO+CCR7+, CM), naïve (CD45RA+CD45RO-CCR7+), double positive (CD45RA+CD45RO+) and double negative (CD45RA-CD45RO-). (**B**) Comparison of CD27- and CCR7-based gating. Each dot represents one donor. (**C**) Relative percentages (y-axis) of CD8 T cell subpopulations in patients with mild-to-moderate psoriasis (n=7) compared to healthy subjects (n=11). (**D**) NK, NKT, CD8, and CD8- T cell distribution (pie charts) and mean frequency of Eff, EM, CM, naïve, double positive, double negative and MAIT CD8 T cells (stacked bars) in psoriasis patients compared to healthy donors.


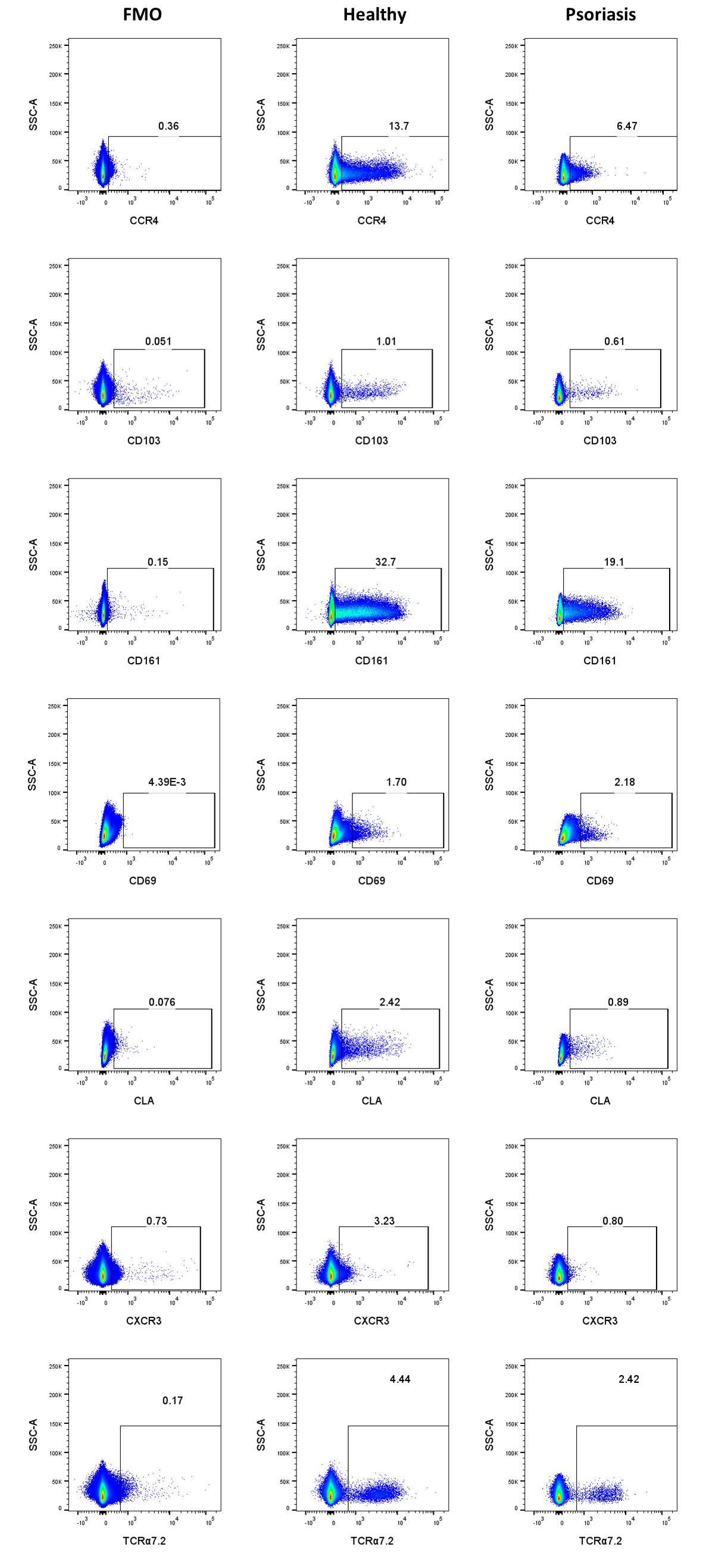


**Supplemental Figure 7. Gating strategy for the determination of positive skin homing markers in unsupervised analyses.** Flow plots of selected cell surface marker expression on CD8 T cells from concatenated files for FMO controls (n=18), healthy donors (n=11) and psoriasis patients (n=7) were generated using FlowJo.


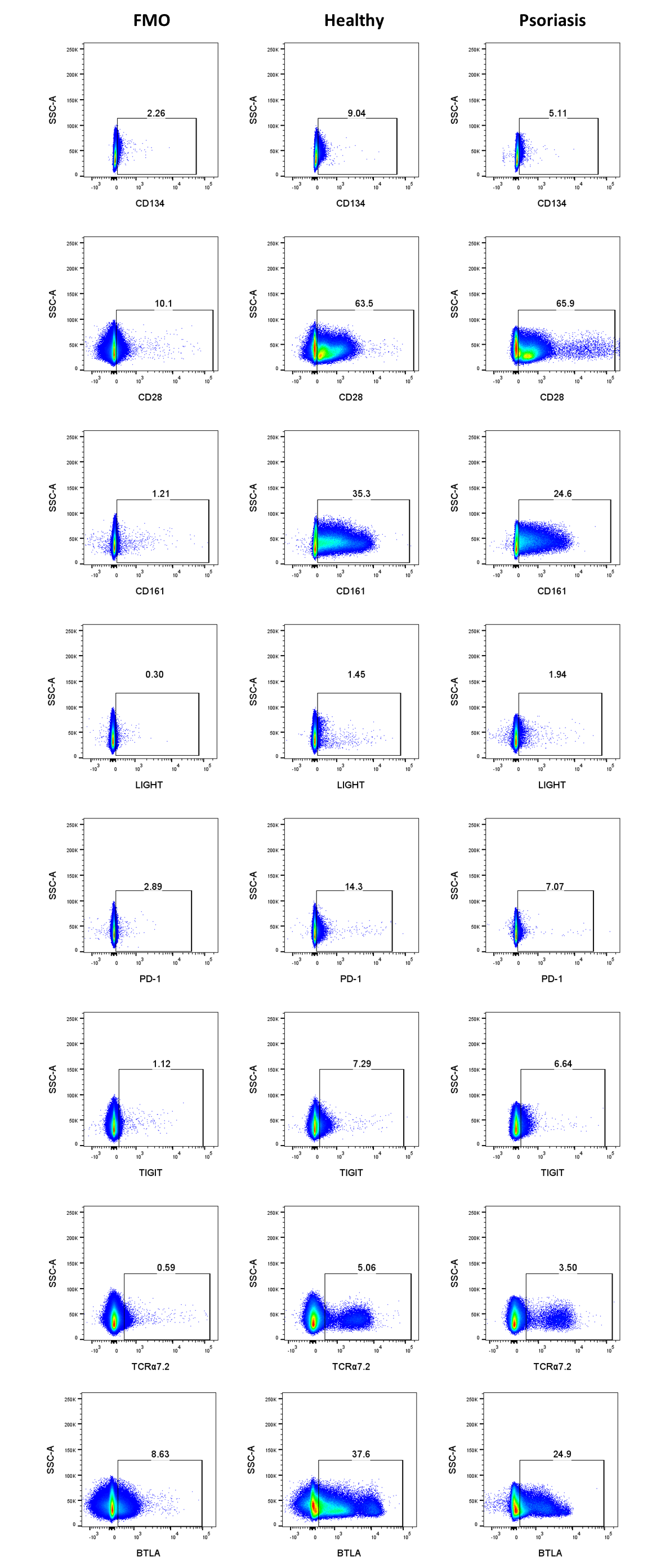


**Supplemental Figure 8. Gating strategy for the determination of positive co-stimulatory/coinhibitory markers in unsupervised analyses.** Flow plots of selected cell surface marker expression on CD8 T cells from concatenated files for FMO controls (n=18), healthy donors (n=11) and psoriasis patients (n=7) were generated using FlowJo.


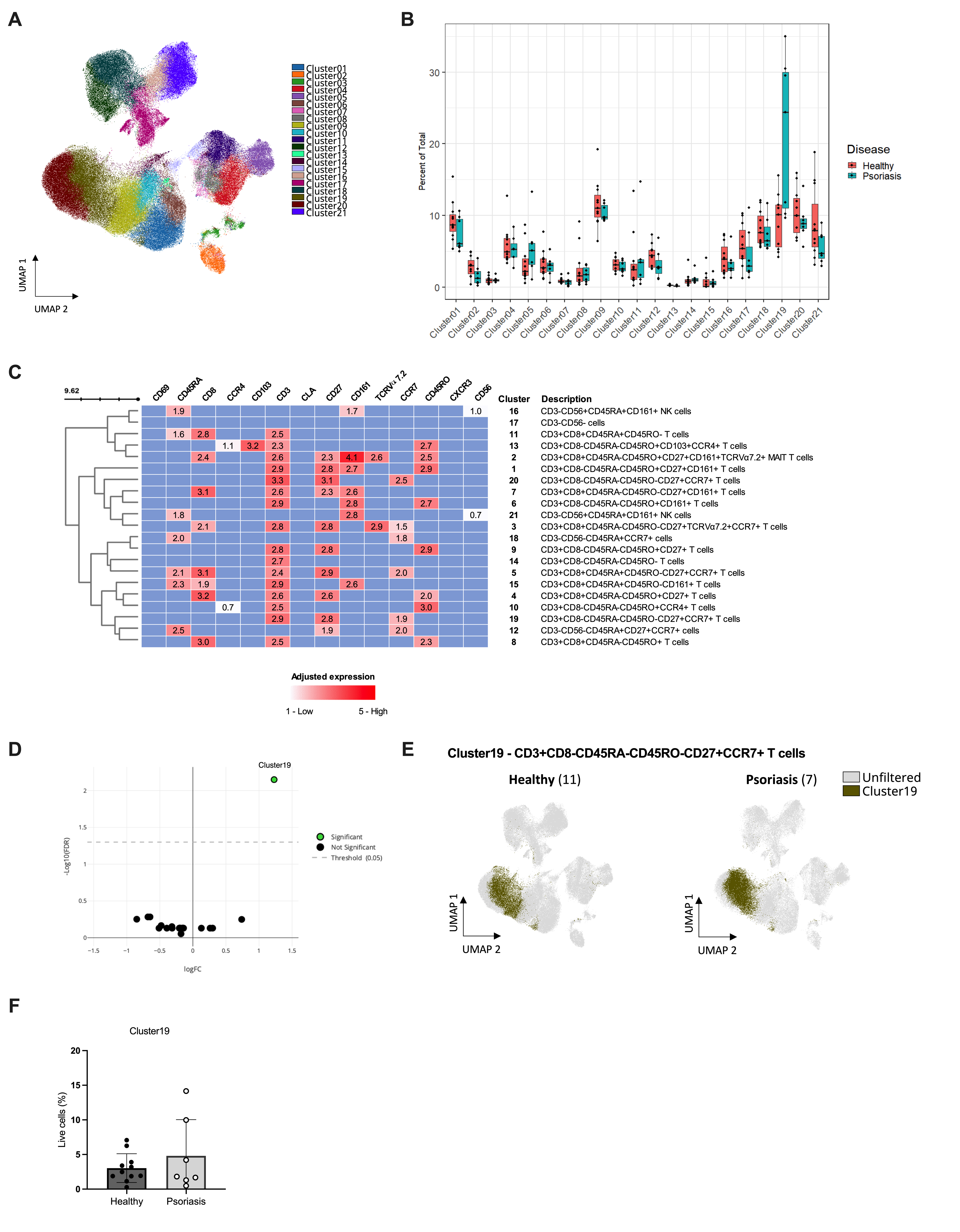


**Supplemental Figure 9. Unsupervised immunophenotype analysis of immune cell subsets of healthy and moderate-to-severe psoriasis patients**. Peripheral blood mononuclear cells (PBMCs) were isolated from patients with mild-to-moderate psoriasis (n=7) and healthy subjects (n=11) and analysed in flow cytometry. (**A**) Identified 21 FlowSOM clusters were projected onto two UMAP dimensions. (**B**) Box plots show the percent abundance (y-axis) of the identified clusters by FlowSOM. (**C**) Heatmap of cell clusters identified by FlowSOM. Rows represent each identified cluster with a description of the phenotype on the right, and columns represent the markers of interest. Adjusted expression values were defined as higher than a specified threshold based on concatenated FMO controls for each marker in flow cytometry. (**D**) Volcano plot displaying the differential count abundance of identified clusters between psoriasis and healthy subjects calculated using EdgeR (**E**) The significant cluster identified in EdgeR (coloured) was overlayed onto not statistically significant clusters (grey) in the UMAP plot Graphs represent mean ± SD. Significant differences were analysed using the Mann-Whitney U test corrected for Benjamini's original FDR method. FDR-adjusted *p* value < 0.05 was considered statistically significant. (**F**) The cluster identified in EdgeR was then analysed using manual gating. Significant differences were analysed using the Mann-Whitney U test.

Abbreviations: FMO, fluorescence minus one control; UMAP, uniform manifold approximation and projection.


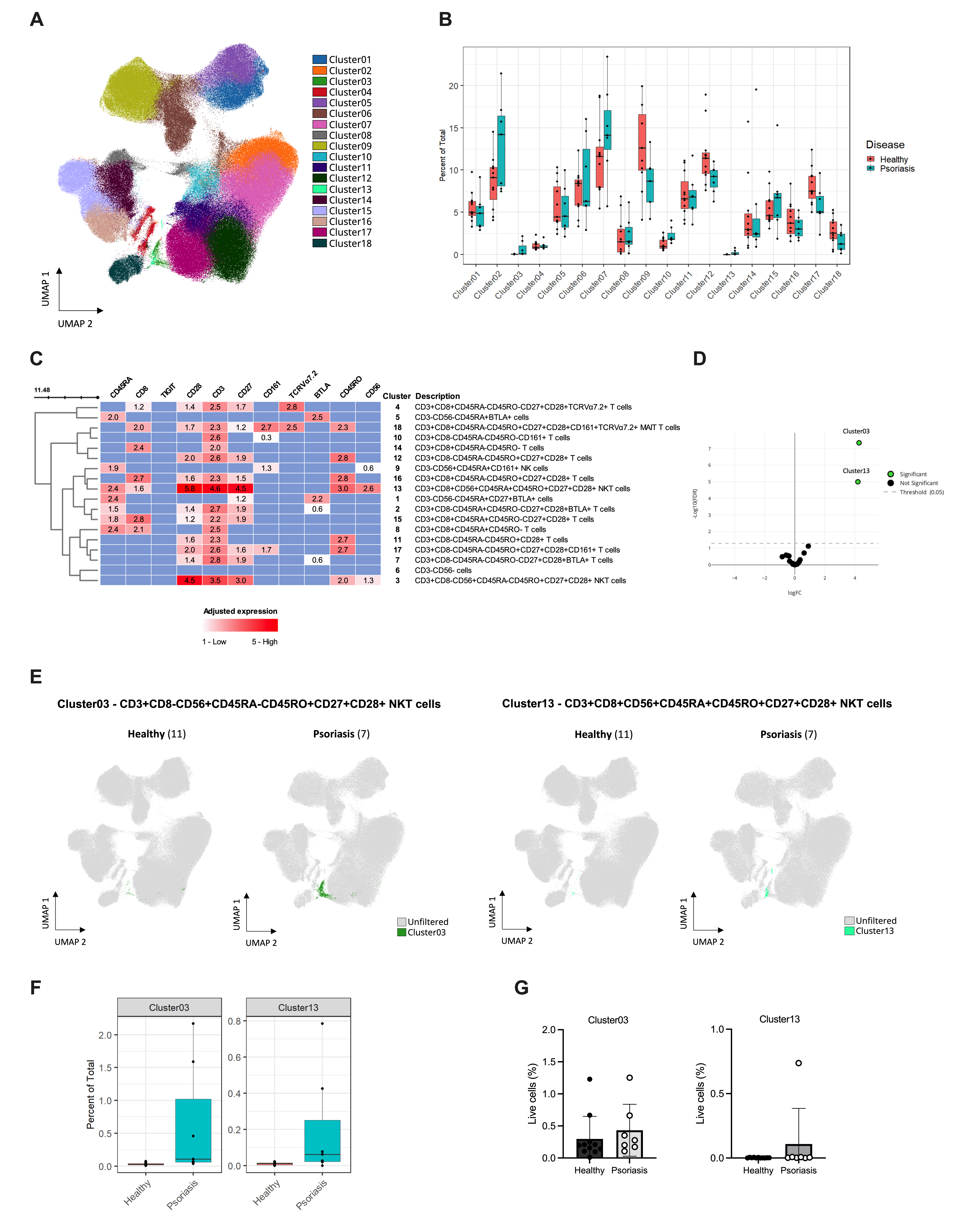


**Supplemental Figure 10. Mild-to-moderate psoriasis patients display increased abundance of NKT cell subsets in unsupervised multi-parametric analyses**. Uniform manifold approximation and projection (UMAP) and FlowSOM were performed in OMIQ in single live lymphocytes isolated from healthy controls (n=11) and patients with mild-to-moderate psoriasis (n=7). (**A**) 18 Identified FlowSOM clusters were projected onto two UMAP dimensions. (**B**) Box plots show the percent abundance (y-axis) of the identified clusters by FlowSOM. (**C**) Heatmap of cell clusters identified by FlowSOM. Rows represent each identified cluster with a description of the phenotype on the right, and columns represent the markers of interest. Adjusted expression values were defined as higher than a specified threshold based on concatenated FMO controls for each marker in flow cytometry. (**D**) Volcano plot displaying the differential count abundance of identified clusters calculated using EdgeR. (**E**) Significant clusters identified in EdgeR (coloured) were overlayed onto not statistically significant clusters (grey) in the UMAP plot. (**F**) Box plots show the percentage relative to the total analysed cells (y-axis) of found clusters using EdgeR. Graphs represent mean ± SD. Significant differences were analysed using the Mann-Whitney U test corrected for Benjamini's original FDR method. FDR-adjusted *p* value < 0.05 was considered statistically significant. (**G**) The cluster identified in EdgeR was then analysed using manual gating. Significant differences were analysed using the Mann-Whitney U test.

Abbreviations: FMO, fluorescence minus one control; UMAP, uniform manifold approximation and projection.

# Supplemental Tables

**Supplemental Table 1**. **Clinical characteristics of patients with mild-to-moderate psoriasis and healthy controls enrolled in the study.**

|  | N. | Females %  (n) | Ethnicity  (%) | Age | | PASI score | |  | Treatment |  |  |
| --- | --- | --- | --- | --- | --- | --- | --- | --- | --- | --- | --- |
|  |  |  |  | *Mean*  *± SD* | *Range* | *Mean*  *± SD* | *Range* | *% topical treatment* | *% on corticosteroid only (n)* | *% on*  *calcipotriol only (n)* |  |
| **Healthy controls** | | 11 | 9.1 (1) | Caucasian  (100) | 50.55 ±  11.99 | 35-69 | n/a | n/a | n/a | n/a | n/a |
| **Psoriasis patients** | | 7 | 42.8 (3) | Caucasian  (100) | 47.28 ±  19.97 | 33-65 | 6.67 ±  1.64 | 4.2-  8.7 | 100 | 14.2 (1) | 0 (0) |

Data are expressed as mean ± SD.

Abbreviations: n/a, not applicable; PASI, Psoriasis Area and Severity Index

**Supplemental Table 2. Flow cytometry markers used in the study.**


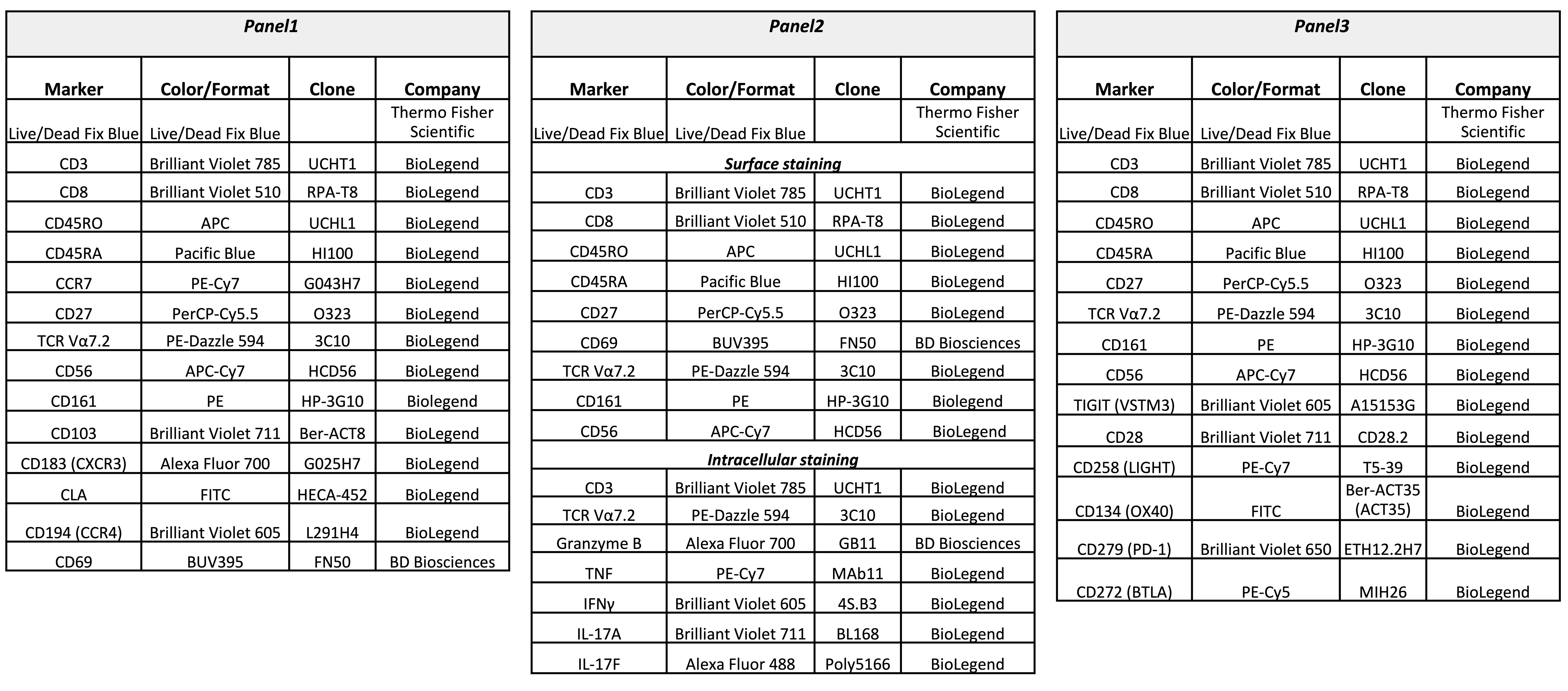


**Supplemental Table 3. Definition of immune cell populations based on the expression of cell surface markers**

| **Cell subtypes** **(gated from lymphocytes)** | **Surface marker** | **Ref** |
| --- | --- | --- |
| NK cells | CD56+CD3- | (1) |
| NKT cells | CD56+CD3- | (1) |
| T cells | CD56-CD3+ | (2) |
| Cytotoxic T cells | CD56-CD3+CD8+ | (3, 4) |
| Memory T cells | CD56-CD3+CD45RO+CD45RA- | (5-8) |
| Central memory T cells | CD56-CD3+CD45RO+CD45RA-CD27+ |  |
|  | CD56-CD3+CD45RO+CD45RA-CCR7+ |  |
| Effector memory T cells | CD56-CD3+CD45RO+CD45RA-CD27- |  |
|  | CD56-CD3+CD45RO+CD45RA-CCR7- |  |
| Naïve T cells | CD56-CD3+CD45RO-CD45RA+CD27+ |  |
|  | CD56-CD3+CD45RO-CD45RA+CCR7+ |  |
| Effector T cells | CD56-CD3+CD45RO-CD45RA+CD27- |  |
|  | CD56-CD3+CD45RO-CD45RA+CCR7- |  |
| MAIT cells | CD56-CD3+TCRVα7.2+CD161+ | (9) |

**Supplemental Table 4. Frequency of lymphocyte and CD8 T cell subsets in patients with mild-**

**to-moderate psoriasis (n=7) and healthy controls (n=11).**


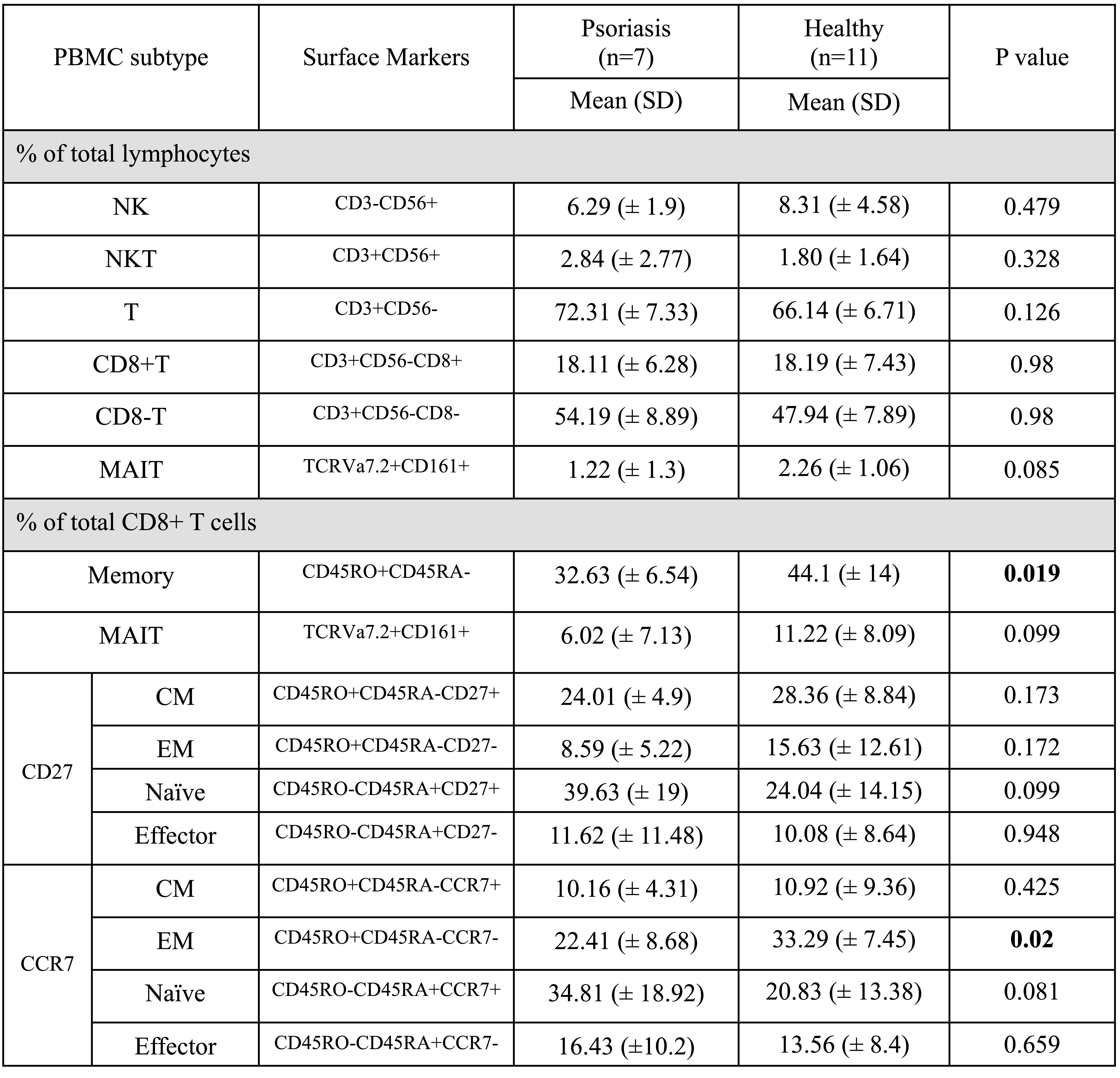


Significant differences were analysed using the Mann-Whitney U test. Statistically significant differences (p value < 0.05) are highlighted in bold.

**Supplemental Table 5. Changes in cluster frequency of stimulated and unstimulated CD3+CD56- T cells in mild-to-moderate psoriasis and healthy**

**subjects.**

|  |  |  |  | **Healthy** | | **Psoriasis** | | **Psoriasis vs Healthy** | | |
| --- | --- | --- | --- | --- | --- | --- | --- | --- | --- | --- |
| **Cluster** | **T cell type** | **Description** | **Stimulation** | **Mean (%)** | **SD** | **Mean (%)** | **SD** | **Change** | **p value** | **FDR** |
| **9** | CD8- | T CD8-CD45RA-CD45RO-CD27+CD161+ cells | Unstimulated | 19.24 | 3.35 | 11.61 | 4.60 | ↓ | 0.005 | 0.008 |
| **11+12** | CD8- | T CD8-CD45RA-CD45RO-CD27+ cells, CD69+ | Unstimulated | 0.45 | 0.58 | 1.98 | 1.32 | **↑** | 0.007 | 0.011 |
| **3** | CD8- | T CD8-CD45RA-CD45RO-CD27+ cells, CD69+TNF+ | PMA/ION | 0.02 | 0.04 | 1.36 | 2.85 | **↑** | 0.001 | 0.004 |
| **7** | CD8- | T CD8-CD45RA-CD45RO-CD27+ cells, IFNγ+TNF^lo^ | PMA/ION | 4.35 | 2.20 | 1.43 | 0.75 | ↓ | 0.007 | 0.011 |
| **11+12** | CD8- | T CD8-CD45RA-CD45RO-CD27+ cells, CD69+ | PMA/ION | 0.07 | 0.08 | 5.34 | 11.13 | **↑** | 0.001 | 0.002 |
| **5** | CD8- | T CD8-CD45RA-CD45RO-CD27+ cells | Anti-CD3/CD28 | 3.03 | 1.92 | 14.08 | 5.00 | **↑** | 0.001 | 0.002 |
| **7** | CD8- | T CD8-CD45RA-CD45RO-CD27+ cells, IFNγ+TNF^lo^ | Anti-CD3/CD28 | 3.82 | 2.19 | 0.79 | 0.56 | ↓ | 0.002 | 0.005 |
| **9** | CD8- | T CD8-CD45RA-CD45RO-CD27+CD161+ cells | Anti-CD3/CD28 | 1.38 | 0.29 | 2.81 | 0.61 | **↑** | 0.001 | 0.002 |
| **10** | CD8- | T CD8-CD45RA-CD45RO-CD27+ cells | Anti-CD3/CD28 | 4.12 | 1.14 | 6.49 | 1.84 | **↑** | 0.007 | 0.022 |
| **13** | CD8- | T CD8-CD45RA-CD45RO-CD27+CD161+ cells,  CD69+Granzyme B^+^ | Anti-CD3/CD28 | 3.68 | 1.05 | 1.64 | 0.73 | ↓ | 0.002 | 0.007 |
| **14** | CD8- | T CD8-CD45RA-CD45RO-CD27+CD161+ cells, CD69+Granzyme B^lo^IL-17A+IL-17F+TNF+ | Anti-CD3/CD28 | 0.54 | 0.13 | 0.19 | 0.21 | ↓ | 0.005 | 0.016 |
| **15** | CD8+ | T CD8+CD45RA+CD45RO-CD27+ cells | Anti-CD3/CD28 | 2.47 | 0.85 | 7.72 | 4.01 | **↑** | 0.005 | 0.016 |

Mann Whitney U calculated between healthy and psoriasis for each stimulation, p values corrected using the Benjamini-Hochberg false discovery rate method (FDR). Arrows represent the relative change from healthy controls. Abbreviations: IFNγ, interferon gamma; PMA/ION, phorbol 12-myristate 13-acetate plus ionomycin; SD, standard deviation, TNF, tumour necrosis factor.

**Supplemental Table 6. Correlation between PASI scores and frequency of identified unsupervised CD8- T cell clusters in psoriasis patients (n=7)**

| **Cluster** | **Condition** | **Description** | **r** | **p value** | **FDR-adjusted p value** | **R^2^** | **p value LR** |
| --- | --- | --- | --- | --- | --- | --- | --- |
| 13 | Ex vivo | T CD8-CD45RA-CD45RO+CD103+CCR4+ cells | -0.847 | **0.0238** | 0.5000 | 0.6793 | **0.0226** |
| 6 | Unstimulated | T CD8-CD45RA-CD45RO-CD27+CD161+ cells, TNF+ | -0.8975 | **0.0143** | 0.2286 | 0.7371 | **0.0134** |
| 2 | Anti-CD3/CD28 | T CD8-CD45RA-CD45RO-CD27+ cells, IFNγ+TNF+ | -0.9364 | **0.0040** | 0.0675 | 0.7825 | **0.0082** |
| 10 | Anti-CD3/CD28 | T CD8-CD45RA-CD45RO-CD27+ cells | -0.7748 | **0.0492** | 0.4183 | 0.6102 | **0.0381** |

In bold p values < 0.05. Abbreviations: IFNγ, interferon gamma; EM, effector memory; FDR, false discovery rate; LR, linear regression; MAIT, mucosal-associated invariant T cells; r, Spearman correlation; TNF, tumour necrosis factor.

## References

1. Koreck A, Suranyi A, Szöny B, Farkas A, Bata-Csörgö Z, Kemeny L, et al. CD3+ CD56+ NK T cells are significantly decreased in the peripheral blood of patients with psoriasis. Clinical & Experimental Immunology. 2002;127(1):176-82.

2. Vavilova JD, Ustiuzhanina MO, Boyko AA, Streltsova MA, Kust SA, Kanevskiy LM, et al. Alterations in the CD56− and CD56+ T Cell Subsets during COVID-19. International Journal of Molecular Sciences. 2023;24(10):9047.

3. Sigmundsdóttir H, Gudjonsson J, Jónsdóttir I, Ludviksson B, Valdimarsson H. The frequency of CLA+ CD8+ T cells in the blood of psoriasis patients correlates closely with the severity of their disease. Clinical & Experimental Immunology. 2001;126(2):365-9.

4. Hu Y, Chen Y, Chen Z, Zhang X, Guo C, Yu Z, et al. Dysregulated peripheral invariant natural killer T cells in plaque psoriasis patients. Frontiers in Cell and Developmental Biology. 2022;9:799560.

5. Bruno M, Davidson L, Koenen HJ, van den Reek JM, van Cranenbroek B, de Jong EM, et al. Immunological effects of anti‒IL-17/12/23 therapy in patients with psoriasis complicated by Candida infections. Journal of Investigative Dermatology. 2022;142(11):2929-39. e8.

6. Herrmann M, Schulte S, Wildner NH, Wittner M, Brehm TT, Ramharter M, et al. Analysis of co-inhibitory receptor expression in COVID-19 infection compared to acute plasmodium falciparum malaria: lAG-3 and TIM-3 correlate with t cell activation and course of disease. Frontiers in immunology. 2020;11:1870.

7. Duraiswamy J, Ibegbu CC, Masopust D, Miller JD, Araki K, Doho GH, et al. Phenotype, function, and gene expression profiles of programmed death-1hi CD8 T cells in healthy human adults. The Journal of Immunology. 2011;186(7):4200-12.

8. Alonso-Arias R, Moro-García MA, López-Vázquez A, Rodrigo L, Baltar J, García FMS, et al. NKG2D expression in CD4+ T lymphocytes as a marker of senescence in the aged immune system. Age. 2011;33:591-605.

9. Salou M, Nicol B, Garcia A, Baron D, Michel L, Elong-Ngono A, et al. Neuropathologic, phenotypic and functional analyses of mucosal associated invariant T cells in multiple sclerosis. Clinical Immunology. 2016;166:1-11.
